# A pH-dependent anti-CD47 antibody that selectively targets solid tumors and improves therapeutic efficacy and safety

**DOI:** 10.1186/s13045-023-01399-4

**Published:** 2023-01-17

**Authors:** Yulu Li, Juan Liu, Wei Chen, Wei Wang, Fang Yang, Ximing Liu, Yao Sheng, Kaixin Du, Miaomiao He, Xueyuan Lyu, Huiyu Li, Linlin Zhao, Zhizhong Wei, Fengchao Wang, Sanduo Zheng, Jianhua Sui

**Affiliations:** 1 Peking University-Tsinghua University-National Institute of Biological Sciences (PTN) Joint Graduate Program, School of Life Sciences, Peking University, Beijing, China; 2 National Institute of Biological Sciences (NIBS), Beijing, China; 3 Graduate School of Peking Union Medical College and Chinese Academy of Medical Sciences, Beijing, China; 4 PTN Joint Graduate Program, School of Life Sciences, Tsinghua University, Beijing, China; 5 Tsinghua Institute of Multidisciplinary Biomedical Research, Tsinghua University, Beijing, China

**Keywords:** CD47, pH-dependent antibody, Solid tumor, Tumor selectivity, Side effects, Therapeutic efficacy, Fc effector function

## Abstract

**Background:**

The antiphagocytic molecule CD47 is overexpressed in a wide variety of cancer cells, and antibodies targeting CD47 for cancer therapies are currently under intensive investigation. However, owing to the ubiquitous expression of CD47 on healthy cells, anti-CD47 therapies often achieve only weak therapeutic benefits and can induce severe side effects. Here, we report the generation of a pH-dependent anti-CD47 antibody (BC31M4) which selectively binds to tumors under the acidic solid tumor microenvironment.

**Methods:**

BC31M4 was generated using antibody phage display and a pH-dependent selection strategy. The pH-dependent binding and blocking activities of BC31M4 were verified using in vitro assays, and the structural basis of the pH-dependent binding property was characterized. BC31M4’s antitumor effect was confirmed by both phagocytosis assays and studies in xenograft models. The tumor selectivity, mechanism of action, PK properties, side effects, and therapeutic efficacy were further evaluated in humanized (hCD47 and its receptor hSIRPα) immunocompetent syngeneic mouse models.

**Results:**

The crystal structure reveals that two histidines locate within the CDRs of the light chain directly contribute to the pH-dependent binding of BC31M4. BC31M4 promotes macrophage phagocytosis of tumor cells more potently at acidic-pH than at physiological-pH. Our hCD47/hSIRPα humanized syngeneic mouse model results demonstrated that BC31M4 selectively accumulates in tumors but not in normal tissues. BC31M4 causes minimal side effects and exhibits superior PK properties as compared to the other examined anti-CD47 antibodies. When combined with adoptive T cell transfer, BC31M4 efficiently promotes adaptive immune responses against tumors and also induces immune memory. Moreover, we show that BC31M4’s antitumor effects rely on an Fc that mediates strong effector functions.

**Conclusions:**

Our study illustrates that the development of a tumor-selective, pH-dependent anti-CD47 antibody safely confers strong therapeutic effects against solid tumors, thus providing a promising therapeutic strategy to overcome the challenges of anti-CD47 therapy.

**Supplementary Information:**

The online version contains supplementary material available at 10.1186/s13045-023-01399-4.

## Introduction

CD47 is a ubiquitously expressed transmembrane protein that transmits an antiphagocytic “don’t eat me” signal through binding to its inhibitory receptor signal regulatory protein alpha (SIRPα) on myeloid cells [1, 2]. A wide variety of cancer cells have been found to exploit this mechanism to escape innate immune surveillance through overexpression of CD47 [3–6]. Previous studies have shown that targeting the CD47/SIRPα axis with anti-CD47 agents (including antibodies and SIRPα-Fc fusion proteins) promotes the phagocytosis of tumor cells by macrophages in vitro, and inhibits tumor growth in many human tumor xenograft models [7–12]. However, antibodies can only bind to tumor cells which express human CD47 in these immunocompromised xenograft models. In contrast, within immunocompetent hosts, the ubiquitous expression of CD47 on healthy cells, especially on red blood cells (RBCs) and platelets, poses a huge challenge for anti-CD47 antibody-based therapies. On the one hand, antibodies binding with CD47 on healthy cells lead to antigen sink effect and attendant poor pharmacokinetic (PK) properties [13, 14]. On the other hand, antibody binding with CD47 on healthy cells can lead to severe side effects [15–18]. It is therefore possible that i) the antitumor efficacy of anti-CD47 agents might have been overestimated and ii) the likely treatment-related side effects of such therapies might have been overlooked in research using xenograft models.

Several studies in vitro and in xenograft models have demonstrated that additional prophagocytic signals are required to potentiate the antitumor efficacy of anti-CD47 agents following CD47 blockade, including Fc-Fc gamma receptor (FcγR)-mediated effector functions. The antitumor efficacy was very weak when using antibodies having weak or lacking Fc effector function; whereas, CD47 blockade incorporated with Fc that mediates strong effector function substantially promoted the antitumor efficacy [6, 7, 15, 19, 20]. These results suggest that strong Fc effector function can improve the antitumor efficacy of anti-CD47 therapies. However, anti-CD47 antibodies that mediate stronger Fc effector function have been found to induce more severe side effects in non-human primates, even when treated in a low dosage [15]. Additionally, several studies in syngeneic mouse models have suggested that stimulation of adaptive immune responses is required for the antitumor effects of anti-CD47 therapies [21, 22]. Hu5F9 is an anti-CD47 antibody currently in clinical trials, results from its phase I study demonstrated very limited antitumor efficacy in patients with advanced solid tumors [8, 16]. Further, various side effects such as anemia, hemagglutination, and chills were observed in many patients that received anti-CD47 therapies [16–18].

To overcome these dilemmas in developing anti-CD47 therapies, one attractive strategy is improving the tumor selectivity of CD47-targeting agents. It has been demonstrated that tumor cells preferentially rely on aerobic glycolysis to produce energy (Warburg effect) and excrete a lot of H^+^ to acidify the tumor microenvironment [23–25], with pH often in the range of 6.4–6.8 for solid tumors, such as glioblastoma, colon cancer, melanoma, breast cancer, and lymphoma [26–29], which is obviously distinct from physiological-pH (about 7.4) in normal tissues. Thus, we hypothesize that if an antibody binds strongly to CD47 only under acidic conditions, it should selectively bind to CD47 in solid tumors.

Here, we describe the generation of a pH-dependent anti-CD47 antibody (BC31M4) using antibody phage display technology and a pH-dependent selection strategy. BC31M4 binds to CD47 and blocks the CD47-SIRPα interaction with higher efficiency at acidic-pH than at physiological-pH; accordingly, BC31M4 more potently promotes macrophage phagocytosis of tumor cells at acidic-pH than at physiological-pH in vitro, which still requires the Fc-mediated effector functions. Further, BC31M4 selectively accumulates to solid tumors rather than to normal tissues in humanized syngeneic mouse models. Compared to the other tested anti-CD47 antibodies, BC31M4 causes minimal toxicity and exhibits superior PK properties. When converted into an isotype that mediates strong Fc effector function, BC31M4 in combination with adoptive T cell transfer efficiently enhances the antitumor responses of the adaptive immunity in syngeneic mouse models. Thus, our development of a tumor selective, pH-dependent antibody reconciles therapeutic efficacy with safety to support anti-CD47 therapies against solid tumors.

## Methods

### Cell lines

CHO, Raji, Jurkat, EL4, B16, CT26, MDA-231, and A20 cells were from the Cell Bank of Type Culture Collection (Chinese Academy of Sciences) or ATCC; the FreeStyle 293F were from Life Technologies; the LL/2 cell line was provided by Dr. Li (Beigene); the E.G7 cell line (a derivative of EL4 that expresses OVA) was provided by Dr. Chen (NIBS); the L929 cell line was provided by Dr. Li (NIBS). The CHO-hCD47, E.G7-hCD47, LL/2-hCD47, B16-hCD47, EL4-hCD47, A20-hCD47, and CT26-hCD47 stable cell lines were established by stably expressing full-length human CD47. The 293F-GnTI^−^ cell was generated by knocking out the GnTI gene from the 293F cell using the CRISPR/Cas9 system. All cells were cultured in the recommended conditions (or following the providers’ instructions).

### Expression and purification of proteins

The extracellular domain of CD47 (CD47-ECD) was fused to a His(× 6)-Avi-tag, the fusion protein was produced by transient transfection of FreeStyle 293F cells and purified by affinity chromatography. The extracellular domain of SIRPα was fused to the Fc of mouse IgG2a (mIgG2a); the fusion protein was produced by transient transfection of FreeStyle 293F cells and purified by affinity chromatography, after which the purified protein was further biotinylated (bio-SIRPα-Fc) using a biotinylation kit (Thermo Scientific). The full-length IgG antibodies were produced similarly as previously described [30]. Briefly, the coding sequences of the variable regions of heavy chain (HC) and light chain (LC) were subcloned into corresponding vectors for expressing heavy chains and light chains of human IgG1 (hIgG1), mouse IgG1 (mIgG1), or mouse IgG2a (mIgG2a) isotypes, separately. Antibodies were subsequently expressed by transient transfection of 293F with HC + LC, and purified by protein A or protein G affinity chromatography. The isotype control antibodies (Ctrl. isotype) were specific to a known irrelevant target and were expressed and purified similarly as testing antibodies. The BC31M4-F(ab′)_2_ fragment was generated by pepsin (Sigma) digestion of BC31M4-hIgG1 at pH 3.6, and subsequently purified with anion-exchange chromatography and size-exclusion chromatography.

### pH-dependent selection and optimization of anti-CD47 antibodies

A human non-immune antibody phage display library was used for panning [30]. The CD47-ECD protein used for panning was biotinylated by BirA ligase first and then captured on streptavidin-conjugated magnetic M-280 Dynabeads (Life Technologies); the magnetic beads were incubated with phage-displayed single chain antibodies (phage-scFvs) prepared from the library in pH 6.0 buffer for binding, and bound phages were eluted by pH 7.4 buffer; TG1-*E. coli* cells were transformed with the eluted phages for ampicillin resistance screening, and subsequently rescued for the next round of panning. After two rounds of panning, single clones were picked and produced as phage-scFv form for enzyme-linked immunosorbent assay (ELISA) analysis, or converted into full-length hIgG1 isotype for SPR or flow cytometry analysis. During the antibody optimization, random mutations were introduced into both the third complementarity-determining region (CDR3) of the heavy chain (HCDR3) and the light chain (LCDR3) to construct phage display sub-libraries. These sub-libraries were subsequently screened using the pH-dependent selection strategy described above.

### ELISA

The ELISA binding assays followed a previously described method [30]. Briefly, CD47-ECD was coated on 96-well plates (MaxiSorp, Nunc). For analyzing phage-scFvs, phage-scFvs were added to the CD47-ECD-coated plates, and the binding of phage-scFvs to CD47-ECD was subsequently detected using a mouse anti-M13-HRP antibody (GE Healthcare). For analyzing full-length IgGs, serially diluted IgGs were added, and the binding of IgGs to CD47-ECD was subsequently detected using a mouse anti-human IgG Fc-HRP antibody (Thermo Scientific). These assays were performed in buffers of different pH. Specifically, for testing phage-scFvs during pH-dependent selection, the assays were performed in pH 6.0 and 7.4; for testing phage-scFvs during site saturation mutagenesis of histidines, the assays were performed in pH 6.5 and 7.4; for testing IgGs during binding confirmation, the assays were performed in pH 6.8 and 7.4.

### Binding kinetic analysis by surface plasmon resonance (SPR)

Kinetic analyses of antibody binding to CD47-ECD were measured with a Biacore T200 instrument (GE Healthcare) at 25 °C. Anti-human IgG was immobilized on a CM5 sensor chip using a Human Antibody Capture kit following the manufacturer’s instructions (GE Healthcare). All antibodies analyzed were in hIgG1 form, and captured at similar levels on the chip. Twofold serially diluted CD47-ECD was injected over the surface of the chip. This experiment was performed at pH 7.4 and pH 6.8 buffers. The binding kinetic parameters were determined by fitting the sensograms to a 1:1 binding model using BIAcore T200 evaluation software.

### Flow cytometry-based binding and blocking assays

For the binding assays, serially diluted antibodies (in hIgG1 form) were incubated with CHO-CD47 or tumor cells, and the binding of antibodies to the cells was detected using a goat anti-human IgG-FITC antibody (Thermo Scientific). The binding activity of antibodies is shown as the percentage of binding by normalizing the binding at the highest concentration as 100% binding. For the blocking assays, serially diluted antibodies were incubated with CHO-CD47 or tumor cells in the presence of bio-SIRPα-Fc, and the binding of bio-SIRPα-Fc to the cells was detected using streptavidin-FITC (Sigma). The blocking activity of antibodies is shown as the percentage of inhibition by normalizing the value of ‘bio-SIRPα-Fc only’ as 0% inhibition. Specifically, for blocking analysis of BC27, the assay was performed at pH 6.0, 6.5, 6.9, and 7.4; for binding and blocking analysis of BC31M4 and BC31M5, the assay was performed at pH 6.8 and 7.4. Specimens were analyzed by a flow cytometry instrument (BD, LSR II).

For the measurement of antibody binding to primary human T cells, human peripheral blood mononuclear cells (PBMCs) were incubated with serially diluted antibodies (in hIgG1 form), and the binding of antibodies to the cells was detected using the goat anti-human IgG-PE antibody (Thermo Scientific). Anti-CD8 (clone SK1, Biolegend), anti-CD4 (clone OKT4, Biolegend), and anti-CD3 (clone SK7, Biolegend) were used to identify T cells in PBMCs. The assay was performed at pH 6.8 and 7.4 separately. T cells were defined as follows: CD4^+^ T cells: CD3^+^CD4^+^, CD8^+^ T cells: CD3^+^CD8^+^. Specimens were analyzed by flow cytometry.

### Crystallization and solving of the BC31M5-CD47 complex structure

The Fab of BC31M5 (with a heavy chain C-terminal His(× 6)-tag) was expressed by transiently transfection of HEK293 cells. The human CD47-ECD (residues 1–118) with a C15G mutation and a C-terminal His(× 6)-tag was expressed by transiently transfection of Expi293F-GnTI^−^ cells. The secreted BC31M5 and CD47 proteins were separately purified by Ni–NTA chromatography (Qiagen). BC31M5 and CD47 were mixed at ratio of 1:1.2 in pH 6.0 HBS (10 mM HEPES pH 6.0, 150 mM NaCl). The BC31M5-CD47 complex was purified using a Superdex S200 column (GE Healthcare), and was concentrated to 10 mg/mL for crystallization. Crystals were obtained by addition of proteins to an equal volume of 0.2 M Zinc acetate dihydrate, 0.1 M Sodium cacodylate trihydrate pH 6.5, 18% w/v PEG 8000. The diffraction data were collected at the Shanghai Synchrotron Radiation Facility (BL17B) and, integrated and scaled using XDS [31]. The crystals were of the P2_1_2_1_2_1_ space group, and were solved by molecular replacement with Phaser using the crystal structures of CD47 (PDB ID 5TZT) and Fab (PDB ID 4JPK) as search models. Two closely related complexes were found in the asymmetric unit, and the model was iteratively built in Coot [32] and refined in PHENIX [33].

### Mice

NOD-SCID and BALB/c mice were purchased from Vital River. C57-hCD47/hSIRPα mice were generated as previously described [34], and BALB/c-hCD47/hSIRPα mice were generated using the same strategy. Briefly, using CRISPR/Cas9 gene editing method, genes (exon 2 of *CD47* and exon 2 of *SIRP*α) coding the IgV domains of both CD47 and SIRPα—which are responsible for the CD47-SIRPα interaction—were replaced with the corresponding orthologous human sequences. OT-I transgenic mice were provided by Dr. Chen (NIBS). All mice were maintained and bred under SPF conditions. All animal experiments were conducted following the National Guidelines for the Housing and Care of Laboratory Animals in China and performed under approved IACUC protocols at NIBS, Beijing.

#### Antibody-dependent cellular phagocytosis (ADCP)

Bone marrow-derived macrophages (BMDMs) from C57-hCD47/hSIRPα mice were used as effector cells in this assay. To prepare BMDMs, mouse bone marrow cells were collected from the tibia and femurs of C57-hCD47/hSIRPα mice, the cells were subsequently stimulated by adding L929-cell-culture-supernatants (containing granulocyte macrophage-colony-stimulating factor (GM-CSF) that secreted by L929 cells) to the medium, and cultured on a 24-well tissue culture plate for 7 days. Tumor cells were labeled with carboxyfluorescein succinimidyl ester (CFSE) following the manufacturer’s instructions (Thermo Scientific), and used as target cells. The BMDMs were labeled with anti-mouse F4/80-Alex Fluor647 (Thermo Scientific) prior to incubation with tumor cells. The CFSE-labeled tumor cells were incubated with different antibodies at room temperature for 15 min and then added to the labeled BMDMs using an effector-to-target ratio of about 1:1. Cells were incubated at 37 °C for 2 h in RPMI1640 medium supplemented with 10% heat-inactivated FBS. During the phagocytosis assay, the pH of the medium was adjusted to pH 7.4 and 6.8 using HEPES and PIPES, respectively. The phagocytosis of tumor cells by macrophages was measured via confocal microscopy.

### Tumor models

For human tumor xenograft models, 6–8 weeks old female NOD-SCID mice were inoculated subcutaneously (*s.c.*) with 1 × 10^6^ Raji cells on the right lower flank. When tumors reached about 50 mm^3^, mice were intraperitoneally (*i.p.*) injected with anti-CD47 antibodies (10 mg/kg, hIgG1 isotype) or PBS as control started on day 8 after inoculation, 2 doses per week for 3 weeks.

For syngeneic mouse models, 6–8-week-old male and/or female C57-hCD47/hSIRPα or BALB/c-hCD47/hSIRPα mice were inoculated subcutaneously (s.c.) with 5 × 10^5^ E.G7-hCD47, 2 × 10^6^ LL/2-hCD47, 2 × 10^5^ B16-hCD47, 5 × 10^5^ A20-hCD47, or 3 × 10^5^ CT26-hCD47 cells on the right lower flank. In C57-hCD47/hSIRPα syngeneic mouse models testing anti-CD47 antibody treatment alone, mice were *i.p.* injected with antibodies (20 mg/kg, mIgG2a isotype) or PBS as control started on day 6 after tumor inoculation, dosing every 3 days for a total of 4 or 6 doses. In BALB/c-hCD47/hSIRPα syngeneic mouse models adopting anti-CD47 antibody treatment alone, mice were *i.p.* injected with a priming dose of antibodies (1 mg/kg, mIgG2a isotype) or PBS as control on day 1 after inoculation, followed by maintenance doses (10 mg/kg) started on day 3, dosing every 4 days for a total of 5 doses.

In combination therapy that comprised antibodies and OT-I T cells, C57-hCD47/hSIRPα mice were inoculated *s.c.* with E.G7-hCD47 cells on the right lower flank as described above, two antibody administration schedules were adopted: i) mice were *i.p.* injected with a priming dose of antibodies (1 mg/kg, mIgG2a or mIgG1 isotype) or PBS as control on day 3 after inoculation, followed by two maintenance doses (10 mg/kg) on days 5 and 11; ii) mice were *i.p.* injected with a priming dose of BC31M4 (1 mg/kg, mIgG2a isotype) or PBS as control on day 4 after inoculation, followed by four maintenance doses (1 or 10 mg/kg) on days 6, 10, 14, and 16. OT-I T cells (5 × 10^6^) were transfused intravenously (*i.v.*) on day 8. To prepare OT-I T cells, spleen cells from OT-I mice were stimulated by adding IL-2 (3SBio) and OVA_257-264_-peptide (Sigma) containing RPMI1640 medium supplemented with 10% FBS and 0.05 mM 2-mercaptoethanol, followed by culturing and passaging for 4 days before injection.

For the tumor rechallenge experiments, C57-hCD47/hSIRPα mice were inoculated *s.c.* with E.G7-hCD47 cells on the right lower flank as described above, mice were *i.p.* injected with a priming dose of 1 mg/kg BC31M4 or BC31M5 (both have mIgG2a isotype) or PBS on day 3 after inoculation, followed by two maintenance doses (10 mg/kg) on days 5 and 11, OT-I T cells were transfused *i.v.* on day 8. Mice that survived from the combination therapy were inoculated *s.c.* with 5 × 10^5^ E.G7-hCD47 or 2 × 10^5^ EL4-hCD47 cells on the left lower flank, at about 4 months after the initial tumor inoculation. As controls, age-matched naïve C57-hCD47/hSIRPα mice were inoculated with the same tumor cells.

In all tumor models, tumor volumes were calculated using the modified ellipsoid formula (length × width^2^ × π / 6) based on caliper measurements.

### Hematotoxicity analysis

Healthy C57-hCD47/hSIRPα mice (male and female, about 12-week-old) were injected *i.p.* with a single dose of anti-CD47 antibodies (20 mg/kg, mIgG2a isotype) or PBS. Blood was drawn from the retro-orbital plexus and collected in dipotassium-EDTA anticoagulation tubes at 3 h after injection. The hematological analyses were performed using the ADVIA 2120 Hematology System (Siemens) to assess the complete blood count. This analysis was carried out at the Vital River Labs (Beijing).

### In vitro hemagglutination analysis

Antibodies (mIgG2a isotype) were threefold serially diluted in pH7.4 PBS from 600 nM in a U-bottom shaped 96-well tissue culture plate; RBCs from C57-hCD47/hSIRPα mice were resuspended in pH7.4 PBS and added at 1:1 volume ratio to the diluted antibodies (the final RBC density was 6 × 10^6^ cells/well). The plate was incubated at room temperature for 2 h. The RBCs were further diluted and analyzed by a flow cytometry instrument (BD, LSR II); cell aggregation was assessed by the increase of FSC-A and SSC-A values in dot plots, compared to the PBS control.

### Measurement of antibody distribution

C57-hCD47/hSIRPα mice (female) were inoculated with 5 × 10^5^ E.G7-hCD47 cells *s.c.* on the right lower flank as described above. Antibodies (mIgG2a isotype) were labeled with Cy7 NHS Ester (Amersham, GE) following the manufacturer’s instructions. When tumors reached volumes of about 500 mm^3^, mice were *i.p.* injected with a priming dose of antibodies (1 mg/kg) or PBS as control, followed by giving a single maintenance dose (5 mg/kg) two days later. For the in vivo antibody distribution and persistence analysis, mice were monitored by in vivo fluorescence imaging using the IVIS Lumina III Imaging System (PerkinElmer) with excitation at 745 nm and emission measured at 800 nm; measurement was conducted at 3, 24, and 72 h after the maintenance dose. The total radiant efficiency was quantified for in vivo fluorescence imaging. For the ex vivo antibody distribution analysis, tumors and organs (spleens, livers, kidneys, and lungs) were isolated from mice for fluorescence imaging at 3 h and 72 h after the maintenance dose. The average radiant efficiency was quantified for ex vivo fluorescence imaging. The radiant efficiency was quantified using Living Image Analysis Software (PerkinElmer).

### PK analysis

Healthy C57-hCD47/hSIRPα mice were *i.p.* injected with a priming dose of antibodies (1 mg/kg, mIgG2a isotype), followed two days later by a single dose of 20 mg/kg. Blood was collected at different time points (from 15 min to 29 day) after the 20 mg/kg dose. Antibody concentrations in serum were measured by ELISA (in pH6.5 buffer). The PK data were evaluated with WinNonlin software.

### Measurement of body temperature and treatment-related-death

C57-hCD47/hSIRPα mice used in this study were treated with different antibodies adopting different treatment strategies. Side effects in these mice were recorded after antibody treatment, which assessed by monitoring body temperature and treatment-related-death. Mice body temperatures were measured at about 3 h after antibody injections using an infrared thermometer. The treatment-related-death of mice includes the following events: deaths of mice due to the side effects within 24 h after antibody treatment; mice endured continuous temperature drop and lethargy, or disability, for more than 24 h after antibody treatment that were euthanized; mice endured weight loss (more than 20%) and lethargy after antibody treatment that were euthanized. Mice in the repeated experiments (not shown) were included. In the summary of the body temperature of C57-hCD47/hSIRPα mice, 30 mice shown in the PBS group were randomly selected from the total mice using RAND function in Excel. The body temperature was summarized as temperature drop (compared to the average temperature in the corresponding control group). The mice examined for the antibody distribution and PK analyses were excluded from side effect assessment; additionally, mice used in the hematotoxicity analysis were excluded from treatment-related-death assessment.

### Statistical analysis

All statistical analyses were performed using GraphPad Prism. For the data from in vitro experiments, Ordinary one-way ANOVA was used for comparisons of three or more groups, and unpaired Student’s t tests was used for comparisons of two groups. For the data from in vivo experiments, comparisons between the antibody treated groups and the corresponding PBS control were assessed using two-way ANOVA for tumor growth and, log-rank (Mantel Cox) tests for survival; Tumor volumes are shown as mean ± SEM. In all statistical analyses, the *P* values (* *P* < 0.05, ** *P* < 0.01, *** *P* < 0.01, **** *P* < 0.0001) were considered significant.

## Results

### Generation of a pH-dependent anti-CD47 antibody that preferentially binds to CD47 in acidic conditions

**Fig. 1:**
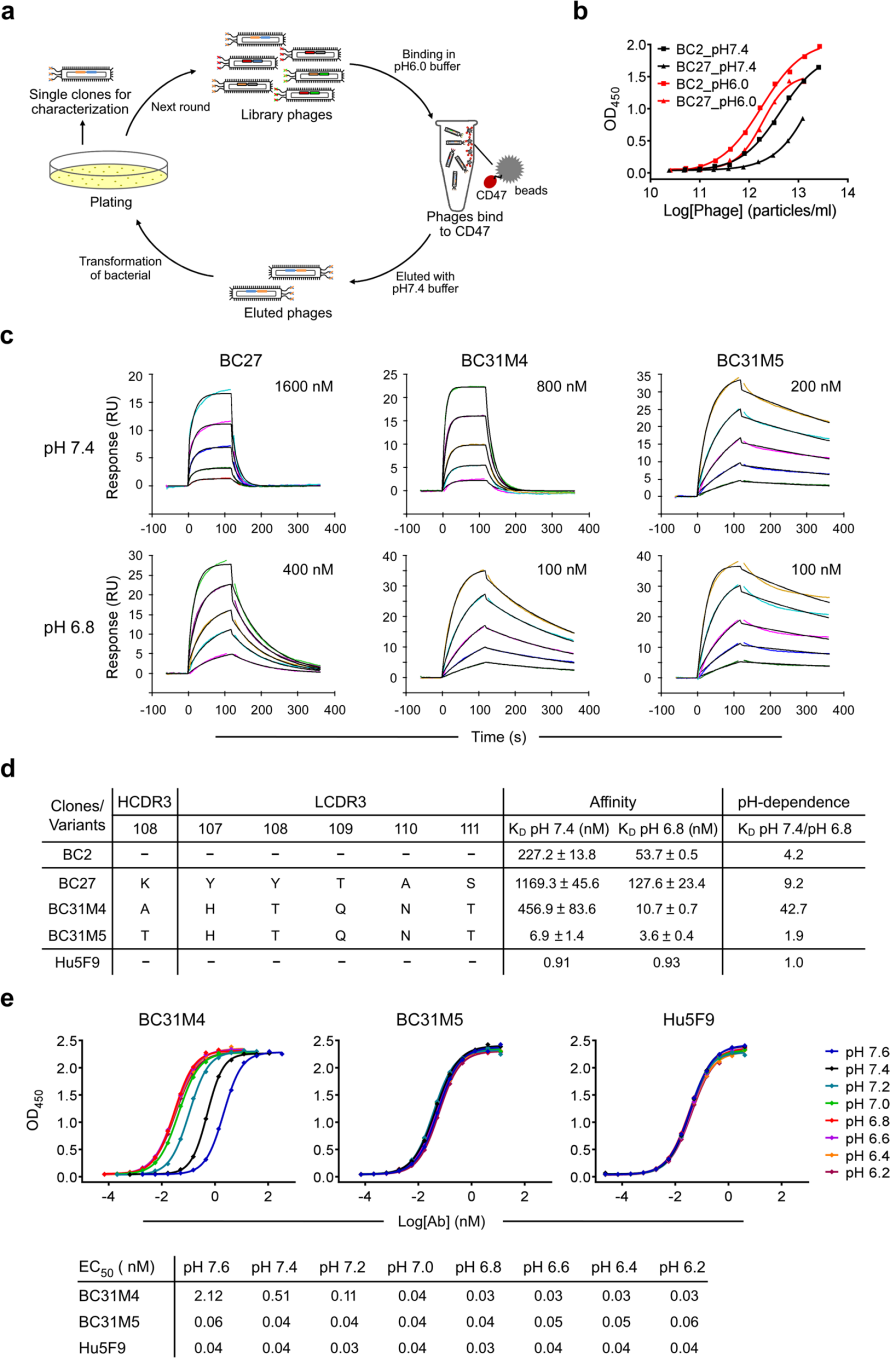
Generation and characterization of pH-dependent anti-CD47 antibodies. **a** Schematic diagram depicting the pH-dependent selection strategy. Library phages were binding to CD47-ECD in pH 6.0 buffer, and subsequently eluted by pH 7.4 buffer; enriched phages were rescued for the next round of panning or, single clones were selected for characterization. **b** Characterization of the pH-dependent binding of BC2 and BC27. Serial dilutions of antibodies (in phage-scFv form) binding to CD47-ECD at pH 7.4 and pH 6.0, measured by ELISA. **c** Representative SPR sensograms of pH-dependent antibodies binding to CD47-ECD at pH 7.4 and pH 6.8; CD47-ECD was diluted from the indicated starting concentrations. Both original sensorgrams (colored lines) and fitting curves (black lines) are shown. **d** Summary of SPR measurements of pH-dependent antibodies, and a non-pH-dependent control Hu5F9; the affinity measurement results (K_D_) shown represent the mean and standard deviation of three replicated experiments. pH-dependence is defined as the ratio of K_D_ at pH 7.4 to pH 6.8. The position of the mutated residues and their corresponding sequences within the HCDR3 and LCDR3 of BC27 (and its variants) are shown. **e** *Top,* serially diluted antibodies binding to CD47-ECD from pH 6.2 to pH 7.6, measured by ELISA. *Bottom,* the calculated EC_50_ for the data of the *top* panel

To identify pH-dependent anti-CD47 antibodies, we implemented a pH-dependent selection strategy to select antibodies from a human antibody phage display library [30]. Library phages were incubated with CD47 in pH 6.0 buffer, and subsequently eluted with pH 7.4 buffer (Fig. 1a). With this panning strategy, phages that bound to CD47 with high affinity at acidic-pH (pH < 7.4) and low affinity at physiological-pH (pH 7.4) were enriched, and two clones that bind to CD47 in a pH-dependent manner (BC2 and BC27) were obtained (Fig. 1b). The binding affinity of the two antibodies to CD47 was measured using SPR at pH 7.4 and pH 6.8: BC27 exhibited relatively higher pH-dependence than BC2, which defined as the ratio of K_D_ at pH 7.4 to pH 6.8, and was therefore selected for further evaluation (Fig. 1c, d and Additional file 1: Fig. S1a). A flow cytometry-based blocking assay showed that BC27 was not able to completely antagonize the CD47-SIRPα interaction at a concentration as high as 1 µM even at acidic-pH condition (Additional file 1: Fig. S1b); this low blocking efficiency might reflect its low binding affinity at acidic-pH.

We next sought to improve both the binding affinity and pH-dependent properties of BC27. Random mutations were introduced into both the CDR3 of the heavy chain (HCDR3) and the light chain (LCDR3) to construct phage display sub-libraries. These sub-libraries were subsequently selected using the pH-dependent strategy described above. After several rounds of intensive optimization-selection, about 15 variants with improved affinity and/or pH-dependence were obtained. Among these variants, BC31M4 (K_D_ = 10.7 nM) had about 12-fold higher affinity than the parental antibody BC27 (K_D_ = 127.6 nM) at pH 6.8, thus achieved a significant increase of pH-dependence from 9.2-fold to 42.7-fold. We also obtained a weak pH-dependent variant BC31M5 that had a pH-dependence of about 1.9-fold (similar affinity to BC31M4 at pH 6.8, much higher affinity at pH 7.4) (Fig. 1c, d). Remarkably, only one amino acid is different between these two antibodies: residue 108 in the HCDR3 is threonine (T) in BC31M5 but alanine (A) in BC31M4.

To further characterize the pH-dependent binding property of BC31M4 and BC31M5, we measured their bindings to CD47 at serial pH conditions (from 6.2 to 7.6) by ELISA. BC31M4 exhibited an apparent pH-dependent binding pattern with 72.5-fold binding increase from pH 6.8 to 7.6 (an EC_50_ of 0.03 nM at pH 6.8 and 2.12 nM at pH 7.6) (Fig. 1e); when the pH was lower than 6.8, the affinity did not increase further. Thus, BC31M4 binds most efficiently with CD47 at pH 6.8 or below, matching with the acidic conditions of most solid tumor microenvironments. In contrast, BC31M5 exhibited slight pH-dependent binding ability. The aforementioned anti-CD47 antibody Hu5F9, which has high binding affinity but no pH-dependence (Fig. 1d and Additional file 1: Fig. S1a), did not exhibit pH-dependent binding ability in these assays. Given their different pH-dependent binding properties, we chose BC31M4 as the therapeutic antibody candidate and BC31M5 as a weak-pH-dependent antibody control for further investigation.

### Histidine residues contribute to the pH-dependent binding of BC31M4

**Fig. 2:**
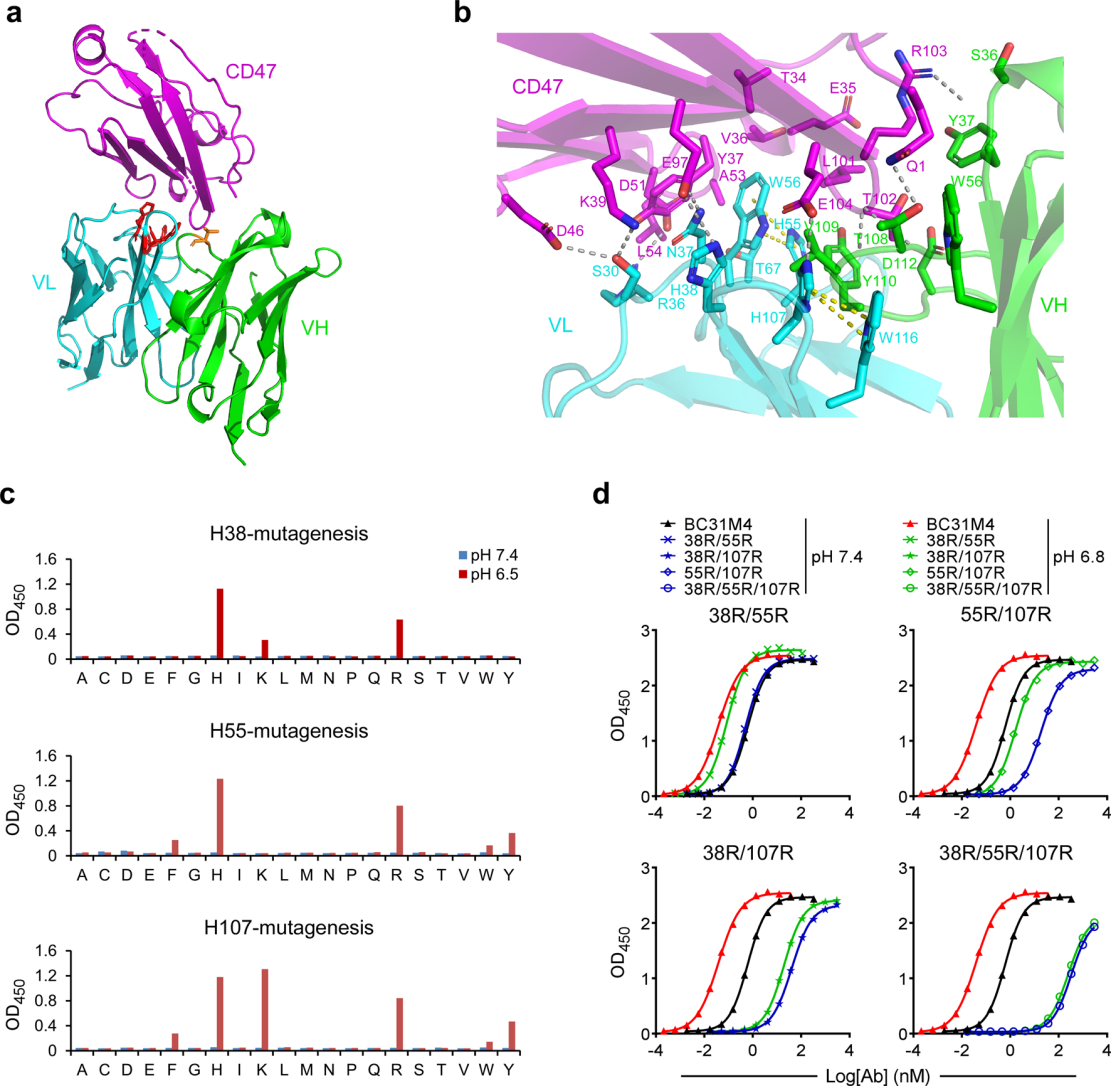
Structural characterization of the pH-dependent binding of BC31M4. **a** Crystal structure of the BC31M5-CD47 complex, depicted as ribbons. T108 (orange) in VH and histidines (red) in VL are shown as sticks. **b** Detailed view of the BC31M5-CD47 interface. Gray dashed lines indicate electrostatic interactions between BC31M5 and CD47. Side chains of contacted residues are shown as sticks. Yellow dashed lines indicate π-contacts. **c** The three histidines (H38, H55, or H107) in the VL of BC31M4 (in phage-scFv form) were mutated into any other amino acids individually (site saturation mutagenesis). These mutants binding to CD47 at pH7.4 and pH 6.5 were measured by ELISA. **d** Serial dilutions of BC31M4 mutants (in hIgG1 form) with the indicated double or triple histidine-to-arginine substitutions binding to CD47-ECD at pH7.4 and pH 6.8, measured by ELISA

To understand the structural basis of this pH-dependent binding property, we solved the crystal structure of BC31M5 (Fab form) in complex with the CD47 extracellular domain at pH 6.5 (2.8 Å resolution; Fig. 2a and Additional file 1: Table S1). Recalling that BC31M4 and BC31M5 have only one amino acid difference, they likely bind to similar epitopes in CD47. The complex structure reveals 15 epitope residues in CD47 at the interaction interface (Table 1). Most of these residues overlap with the previously reported SIRPα binding sites [35], suggesting that these antibodies will maximally antagonize the CD47-SIRPα interaction. In addition, residue T108 in the HCDR3 of BC31M5 forms polar contacts with Q1 and T102 of CD47 (Fig. 2a, b); however, it is expected no similar polar contacts between BC31M4 and CD47 as this residue is A108 in BC31M4.

**Table 1:** Contact residues between BC31M5 and CD47

BC31M5-Fab	HCDR1			HCDR2	HCDR3					LCDR1				LCDR2			LCDR3
	S36	Y37	G38	W55	G107	T108	V109	Y110	D112	S30	R36	N37	H38	H55	W56	T67	H107
CD47	Q1 R103	T102 R103	Q1	Q1	T102	Q1 T102	T102 E104	L101 T102	T102	K39 D46	Y37 D51 L54	Y37 K39 E97	K39 E97	L101	T34 E35 V36 Y37 A53 L101	A53	E104

Contact residues with interatomic distances less than 4 Å are summarized

We next explored how particular amino acids contribute to the pH-dependent binding of BC31M4. The side chain of histidine has an acidic ionization constant (pKa) value of around 6.5 in most proteins [36], this property supports differential antibody binding around this pH range. There are three histidine residues (H38, H55, and H107, defined by IMGT numbering) located within the CDRs of the variable region of light chain (VL). The complex structure shows that H38 (LCDR1) and H107 (LCDR3) form electrostatic contacts with E97 and E104 in CD47, respectively (Fig. 2b and Table 1). These data suggest that H38 and H107 support the pH-dependent binding of BC31M4.

We subsequently conducted site saturation mutagenesis for these histidines individually. The mutagenesis was performed with phage-scFv form, and binding to CD47-ECD was evaluated by ELISA. Many of the mutations dramatically decreased the binding affinity of BC31M4 at pH 6.5. At positions H38 and H107, mutations by replacing with other positively charged amino acids—arginine (R) or lysine (K)—which maintained binding activities comparable to wild-type BC31M4 or retained weak binding activities—confirming that electrostatic contacts formed through protonation of amino acids at these positions promotes BC31M4 binding to CD47. Additionally, at position H55 (LCDR2), a histidine-to-arginine (H-to-R) mutation retained a relatively weak binding activity. And at H55 and H107, mutation to aromatic amino acids—phenylalanine (F), tyrosine (Y), or tryptophan (W)—retained weak binding activity (Fig. 2c). Note that the complex structure shows that H55 and H107 form intra-chain π–contacts with the antibody’s W56 and W116 residues, respectively (Fig. 2b); similar π–contacts may form with mutant variants bearing aromatic (π–π) or positively charged (cation–π) amino acids.

To characterize the contribution of individual histidine to the pH-dependent binding of BC31M4, we generated a set of BC31M4 mutants in which two of the three histidines were substituted with positively charged arginine. These substitutions were performed in hIgG1 form, and binding to CD47 was evaluated by ELISA, with pH-dependent binding assessed as binding deviation between pH 7.4 and pH 6.8. The 38R/55R and 55R/107R substitutions retained pH-dependent binding to CD47 at a similar level to wild-type BC31M4; in contrast, the 38R/107R substitution dramatically diminished the pH-dependent binding; and the 38R/55R/107R substitution completely abrogated pH-dependent binding while also greatly reducing overall binding (Fig. 2d). Collectively, these results support that H38 and H107 are directly involved in the pH-dependent binding between BC31M4 and CD47; whereas H55 contributes minimally to the pH-dependent binding, apparently acting indirectly.

### BC31M4 blocks the cell-surface CD47-SIRPα interaction and promotes macrophage phagocytosis of tumor cells in a pH-dependent manner

**Fig. 3:**
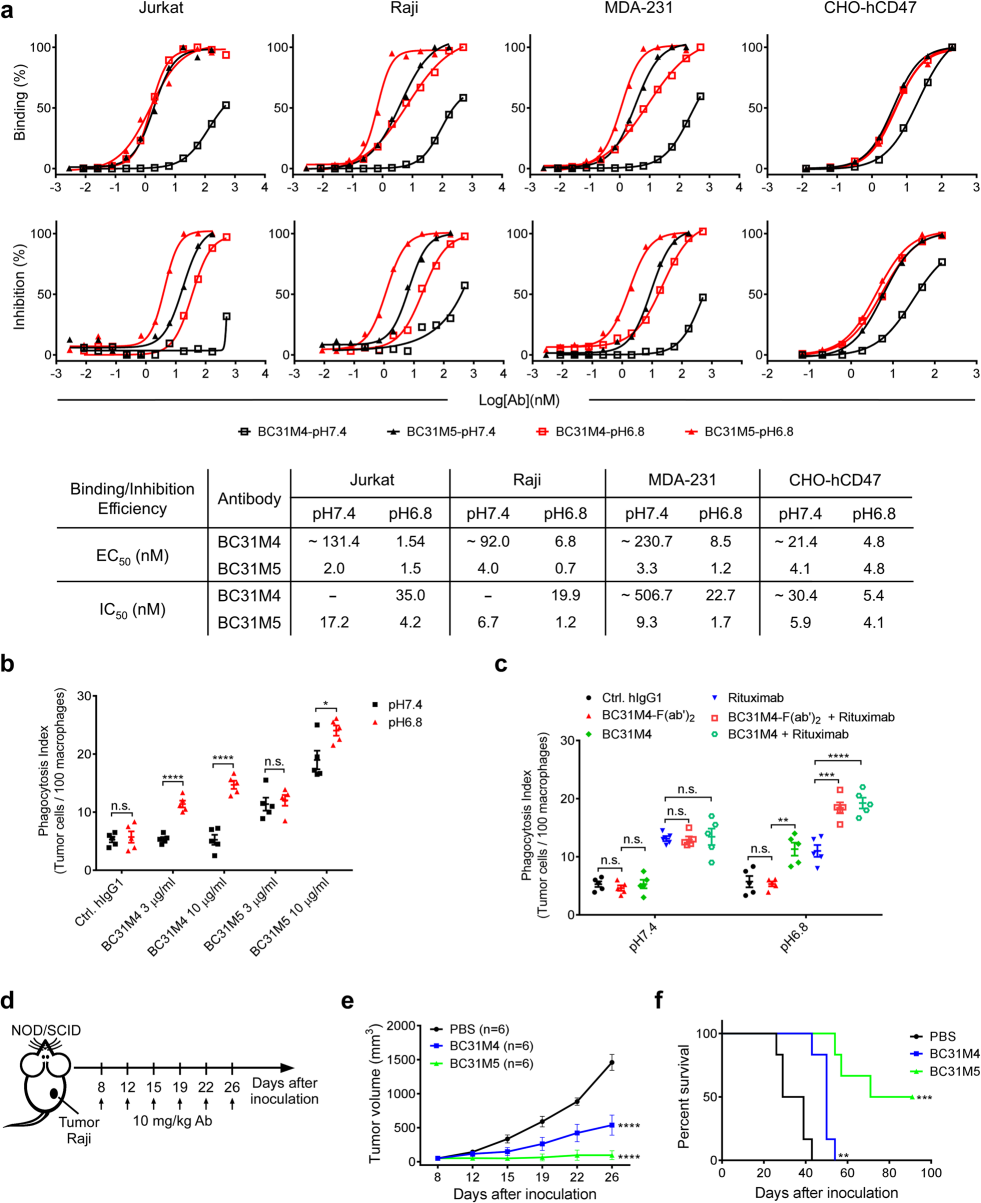
BC31M4 blocks the cell-surface CD47-SIRPα interaction and promotes macrophages phagocytosis of tumor cells. **a** *Top,* the binding and blocking activity of antibodies to different tumor cells at pH 7.4 and pH 6.8. *Bottom,* the calculated EC_50_ and IC_50_ for the data of the *top* panel. “~” indicates estimated values; “-” indicates EC_50_ or IC_50_ values are not measurable. **b, c** Macrophages phagocytosis of Raji cells induced by different antibodies at pH 7.4 and pH 6.8. All antibodies used are of hIgG1 isotype except BC31M4-F(ab′)_2_. Antibody concentration is 1 μg/ml for Rituximab, and 5 μg/ml for BC31M4 and BC31M4-F(ab′)_2_. Phagocytosis index is determined as the number of phagocytosed Raji cells per 100 macrophages. **d** Schematic diagram of tumor inoculation and antibody treatment in human tumor xenograft models. **e** Tumor growth of mice treated as in (**d**). **f** Survival of mice in (**e**). n, number of mice

To determine whether BC31M4 binds to the cell-surface CD47 and blocks the CD47-SIRPα interaction in a pH-dependent manner, a flow cytometry-based binding and blocking assay was performed to evaluate the binding and blocking activity of antibodies to different cells, including a CHO cell line stably expressing full-length human CD47 (CHO-hCD47), Raji (B lymphoma), Jurkat (T lymphoma) and MDA-231 (breast cancer) cells, at pH 6.8 and pH 7.4 conditions separately. Compared to BC31M5, BC31M4 bound to these cells and blocked the binding of SIRPα to these cells with much higher efficiency at pH 6.8 than at pH 7.4 (Fig. 3a). We further investigated the binding of antibodies (BC31M4, BC31M5, and Hu5F9) to human T cells at pH7.4 and pH 6.8. Compared to Hu5F9, both BC31M4 and BC31M5 exhibited pH-dependent binding to CD4^+^ and CD8^+^ T cells in human PBMCs, evidenced by the apparent higher binding efficiency at pH 6.8 than at pH 7.4 (Additional file 1: Fig. S2a).

We next sought to determine the ability of BC31M4 to promote the phagocytosis of tumor cells, an antibody-dependent cellular phagocytosis (ADCP) analysis was performed using Raji cells as targets and bone marrow-derived macrophages (BMDMs) from the C57-hCD47/hSIRPα transgenic mice as effectors. In the C57-hCD47/hSIRPα mice, the IgV domains of both CD47 and SIRPα (responsible for the CD47-SIRPα interaction) are replaced with the corresponding human sequences. Raji cells opsonized with different concentrations of antibodies were incubated with BMDMs at pH 6.8 and pH 7.4 conditions separately; antibodies used here were converted into hIgG1 isotype that mediates strong Fc effector functions in both human and mouse [37–39]. BC31M4 did not induce macrophage phagocytosis of Raji cells at pH 7.4, but significantly promoted phagocytosis at pH 6.8 in a dose-dependent manner. BC31M5 promoted macrophage phagocytosis of Raji cells to a similar extent at pH 6.8 and pH 7.4 (Fig. 3b).

Subsequently, we evaluated the ability of BC31M4-F(ab′)_2_ fragment (lacks Fc) alone or in combination with a tumor-specific monoclonal antibody in inducing phagocytosis. Using Rituximab-hIgG1 (binds to CD20 on Raji cells) as the tumor-specific antibody, Raji cells as target cells, we observed that BC31M4-F(ab′)_2_ in combination with Rituximab induced a significantly higher level of phagocytosis than Rituximab alone did at pH 6.8; however, BC31M4-F(ab′)_2_ alone failed to induce phagocytosis of Raji cells (Fig. 3c).

Additionally, using E.G7-hCD47 cells (a mouse-derived T lymphoma cell line that stably expresses human CD47) as the target cells, BC31M4-F(ab′)_2_ alone or BC31M4-mIgG1 (a mouse isotype with weak Fc effector functions [38, 39]) also did not induce the phagocytosis of E.G7-hCD47, whereas BC31M4-mIgG2a (a mouse isotype with strong Fc effector functions [38, 39]) induced phagocytosis of E.G7-hCD47 cells (Additional file 1: Fig. S2b). Collectively, these results suggest that BC31M4 promotes macrophages phagocytosis of tumor cells requires both blockade of the CD47-SIRPα inhibitory pathway and activation of the Fc-mediated effector functions.

### BC31M4 inhibits tumor growth in human tumor xenograft models

Given that BC31M4 can promote macrophage phagocytosis against tumor cells in vitro, we next examined whether BC31M4 has antitumor effects in vivo using human tumor xenograft models. Raji lymphoma cells were subcutaneously (*s.c.*) inoculated into NOD-SCID mice, and treated with antibodies (in hIgG1 form) intraperitoneally (*i.p.*) (Fig. 3d). Compared to the PBS control, both BC31M4 and BC31M5 significantly inhibited tumor growth and significantly prolonged the survival of mice (Fig. 3e, f). In addition, the antitumor efficacy of BC31M5 was more potent than BC31M4 in these xenograft models, which may be attributed to the higher binding affinity of BC31M5 with CD47, about threefold higher than BC31M4 at pH 6.8 (Fig. 1c, d), albeit with only 1.9-fold pH-dependency. Note that human CD47 expression is absent in these immunocompromised mice, any human CD47-specific antibodies will only bind to tumor cells in these models, regardless of whether they have pH-dependent binding ability or not; and antibodies with higher binding affinity (BC31M5), but not better pH-dependence (BC31M4), are more likely to have better antitumor activity; however, human CD47 is ubiquitously expressed on healthy cells in patients. Therefore, the advantage of BC31M4’s pH-dependent binding cannot be appropriately assessed in these xenograft tumor models.

### BC31M4 selectively accumulates in tumors and exhibits superior PK properties in C57-hCD47/hSIRPα mice

Given that BC31M4 and BC31M5 do not recognize mouse-CD47 (mCD47), xenograft models are unsuitable for evaluating their therapeutic effects in a physiological context wherein CD47 is widely expressed. Thus, and seeking to evaluate the therapeutic efficacy and safety of antibodies in immunocompetent syngeneic mouse models that are more reflective of the CD47 expression profile in humans, we established syngeneic tumor models using the aforementioned C57-hCD47/hSIRPα mice. Because mCD47 does not cross-react with human-SIRPα (hSIRPα) [40], the mouse-derived tumor cell lines were humanized via stable transfection with full-length hCD47 (Cell-hCD47) (Additional file 1: Fig. S3a). Furthermore, to minimize the risk of eliciting anti-drug antibodies in the immune competent C57-hCD47/hSIRPα mouse, antibodies used were converted (unless otherwise noted) into the mIgG2a isotype.

**Fig. 4:**
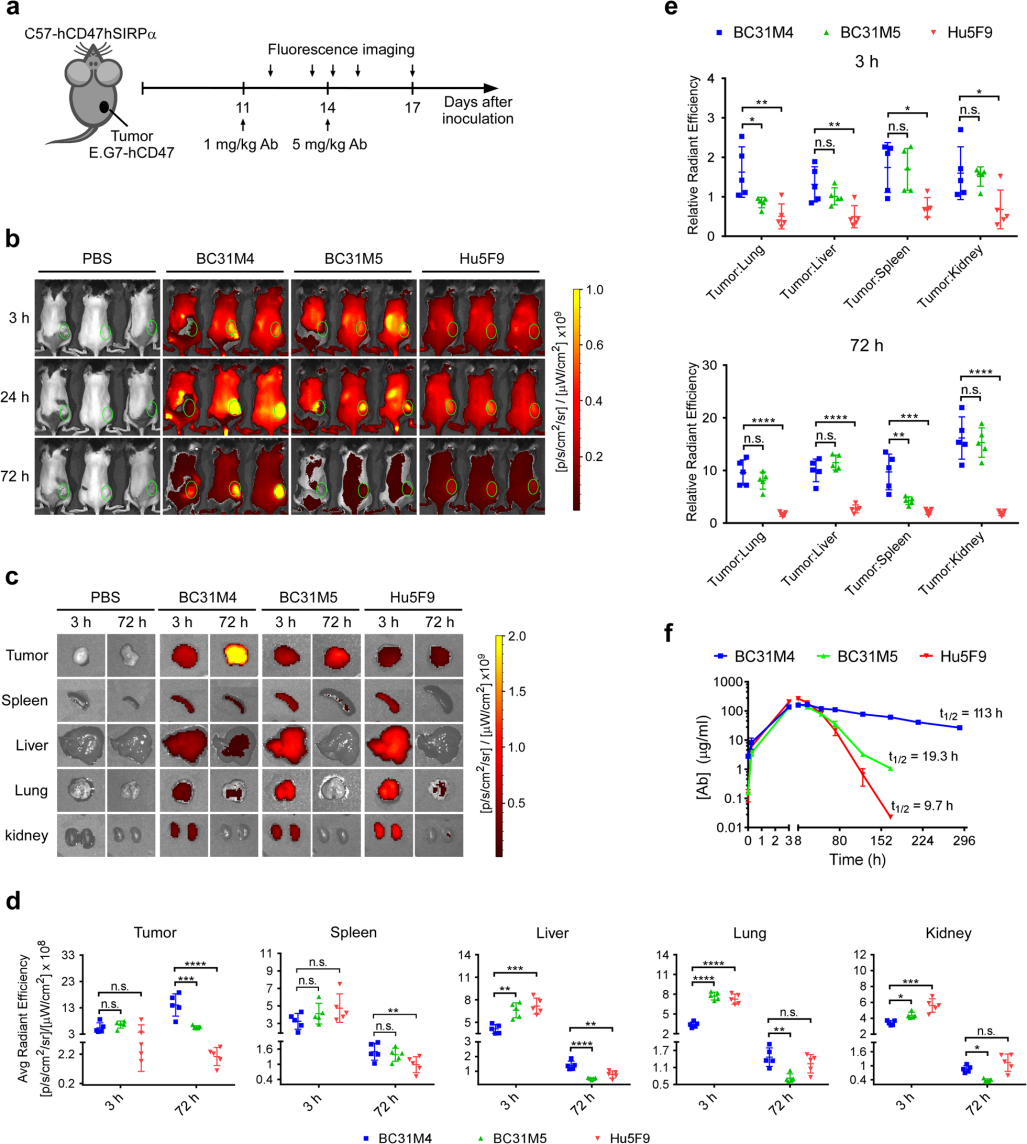
BC31M4 selectively accumulates in tumors and exhibits superior PK properties in C57-hCD47/hSIRPα mice. **a** Schematic diagram showing the tumor inoculation and antibody treatment of mice in (**b, c**). C57-hCD47/hSIRPα mice were *s.c.* inoculated with E.G7-hCD47 cells on the right lower flank. Mice were *i.p.* treated with Cy7-labeled antibodies or PBS. **b** Antibody distribution in mice, monitored by in vivo fluorescence imaging at the indicated time points after the maintenance dose (5 mg/kg). Hair on the back of mice was removed before imaging. Green circles indicate the location of tumors. **c** Antibody distribution in tumors and different organs of mice (n = 5 per group); tumors and organs were isolated for ex vivo fluorescence imaging at 3 h and 72 h after the maintenance dose. Representative images from one mouse in each group are shown. **d** Summary of the antibody distribution in tumors and organs of (**c**), the average quantified radiant efficiency is shown. **e** Summary of the tumor-to-organ fluorescence intensity ratio of (**c**). **f** PK analysis of antibodies in C57-hCD47/hSIRPα mice (n = 2–4 per group). Mice were treated *i.p.* with a priming dose of 1 mg/kg two days before delivery of a single dose of 20 mg/kg. The antibody concentration in serum was monitored, and the half-life of each antibody is indicated

To verify whether BC31M4 selectively binds to tumors in vivo, we measured the biodistribution of antibodies in vivo using whole-body near-infrared fluorescence (NIRF) imaging. Antibodies were labeled with fluorophores (Additional file 1: Fig. S3b). E.G7-hCD47 lymphoma cells were *s.c.* inoculated into C57-hCD47/hSIRPα mice; when tumors reached volumes about 500 mm^3^ (Additional file 1: Fig. S3c), these mice were treated *i.p.* with antibodies. Previous studies showed that adopting a low priming dose before a high maintenance dose (prime-plus-maintenance) can alleviate the side effects of anti-CD47 antibodies in vivo [8]. To reduce the risk of causing severe side effects, antibodies were administered with a priming dose of 1 mg/kg, followed by a single maintenance dose of 5 mg/kg two days later (Fig. 4a). After the priming dose, BC31M4 exhibited more obvious intratumoral accumulation than Hu5F9 and BC31M5 (Additional file 1: Fig. S3d). After the maintenance dose, BC31M4 obviously accumulated at tumor sites at both 24 h and 72 h; in contrast, no similar intratumoral accumulation was observed for BC31M5 and Hu5F9 (Fig. 4b).

We next profiled the antibody distribution in different organs and tissues. Additional mice given the same treatment as above were euthanized at 3 h or 72 h post maintenance dose, tumors and organs (spleen, liver, kidney, and lung) were isolated for fluorescence imaging and quantification of the fluorescence intensity (Fig. 4c, d and Additional file 1: Fig. S2e). Comparing the fluorescence intensities in tumors showed that the BC31M4 signal significantly increased between the 3 h and 72 h sampling time points, whereas the BC31M5 signal remained at a similar level and the Hu5F9 signal appeared to decrease slightly between these time points. The organ analysis showed that the fluorescence intensity of all antibodies was markedly decreased between the 3 h and 72 h sampling time points for all examined normal organs. At 3 h, all antibodies exhibited similar fluorescence intensity in tumors; whereas compared to BC31M5 and Hu5F9, the BC31M4 signal was slightly lower (not significant) in spleen, and significantly lower in other normal organs. At 72 h, the BC31M4 signal was significantly higher than BC31M5 and Hu5F9 in tumors; whereas in normal organs, comparing to BC31M5, the BC31M4 signal was significantly higher in liver, lung, and kidney, but not in spleen; comparing to Hu5F9, BC31M4 signal was significantly higher in spleen and liver, but not in lung and kidney (Fig. 4c, d).

To better determine the extent of antibody accumulation in tumors relative to that in normal tissues, we subsequently calculated the fluorescence signal intensity ratio of the tumor to each of the other organs (tumor-to-organ ratio). Compared to Hu5F9, BC31M4 exhibited relatively high intratumoral accumulation (tumor-to-organ ratio > 1) at 3 h based on all the evaluated organs, and this accumulation became more substantial (tumor-to-organ ratio > 9) at 72 h (Fig. 4e). Additionally, although the pH-dependent binding property of BC31M5 is poor, it also exhibited some level of intratumoral accumulation but significantly lower than BC31M4 especially in tumor-to-lung ratio at 3 h and tumor-to-spleen ratio at 72 h. These results support that pH-dependent binding endows antibodies with selective binding capacity for tumor cells while sparing the healthy cells.

Poor PK due to antigen sink effect is a concern for anti-CD47 antibodies [8]. The weak binding of BC31M4 to normal tissues should reduce antigen sink effect and improve its PK properties. We therefore performed a single-dose PK analysis of antibodies using C57-hCD47/hSIRPα mice. BC31M4 exhibited much better PK properties with a half-life of 113 h, compared with 19.3 h for BC31M5 and 9.7 h for Hu5F9 (Fig. 4f). Collectively, these results support that BC31M4’s pH-dependent binding property endows it with superior PK properties in immunocompetent syngeneic mouse models. It is worth noting that the PK profiles of the three antibodies suggest that the higher accumulation of BC31M4 in most normal organs at 72 h (Fig. 4c, d) is likely due to its higher serum concentration, but not its binding to healthy cells.

### BC31M4 causes minimal side effects in C57-hCD47/hSIRPα mice

**Fig. 5:**
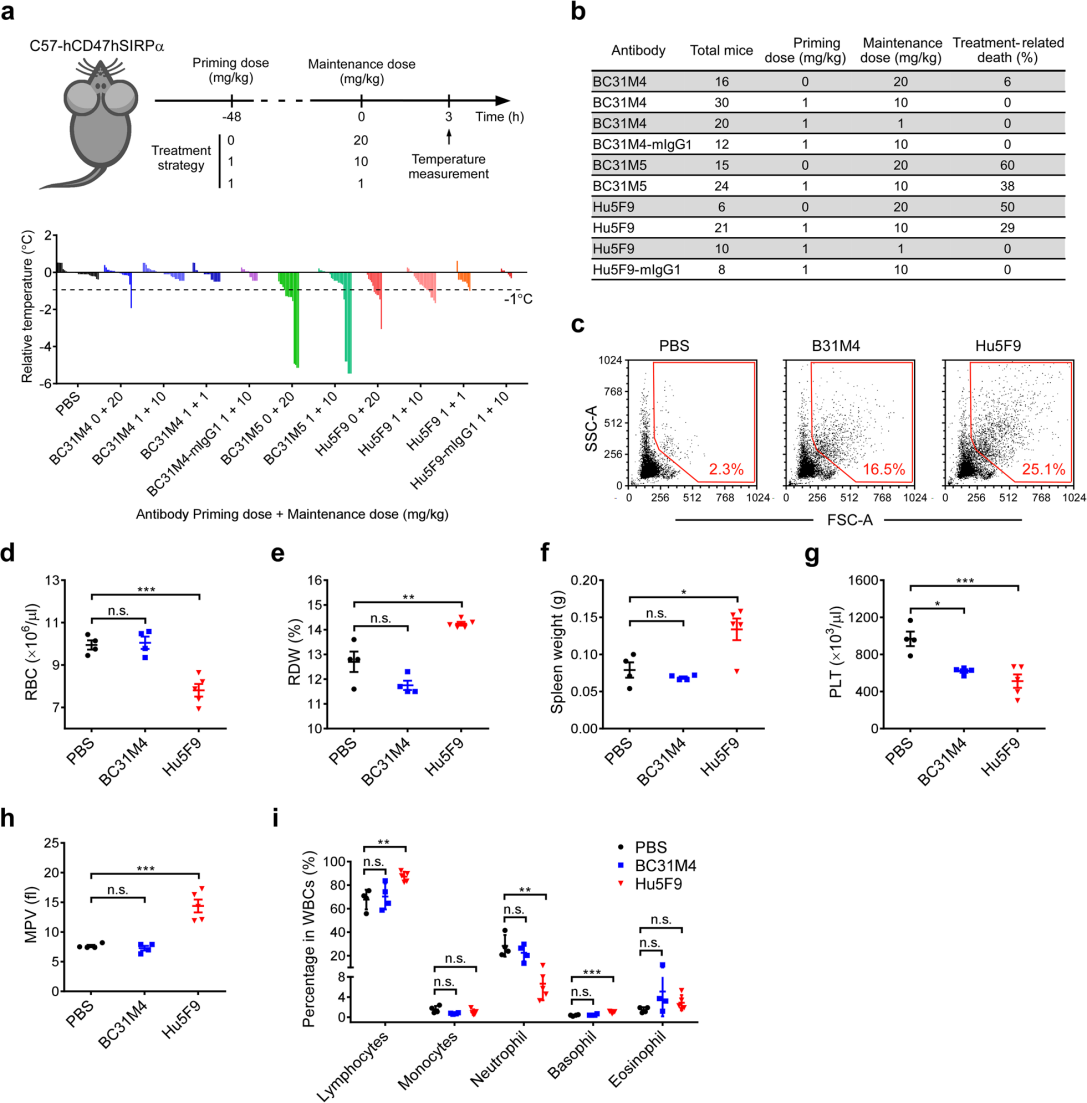
BC31M4 causes minimal hematotoxicity in C57-hCD47/hSIRPα mice. **a** *Top,* schematic diagram of mouse treatment and body temperature measurement. ‘0’ indicates no priming dosing. *Bottom,* body temperature changes compared to the average temperature in the corresponding control group; each histogram represents one mouse. **b** Summary of the treatment-related-death of the antibody treated mice. **c** Hemagglutination test of antibodies in vitro. RBC aggregation was analyzed by flow cytometry. Dot plots of the FSC-A and SSC-A values are shown; percentage of events in the gate (red frame) is denoted. **d, e, g–i** Summary of the complete blood count test of antibody-treated mice. The parameters of RBC count (**d**), red cell distribution width (**e**), platelet count (**g**), mean platelet volume (**h**), and the proportion of individual leukocytes in WBCs (**i**) are shown. **f** Spleen weight of mice used in the complete blood count test. Antibodies used in (**a–f**) are mIgG2a isotype unless otherwise noted

Various side effects have been reported for anti-CD47 therapies in pre-clinical and clinical studies [8, 16–18]. We next adopted different treatment strategies to compare the safety of our antibodies in vivo (Fig. 5a). Briefly, a body temperature drop was the most commonly observed symptom in treated C57-hCD47/hSIRPα mice, which often occurred around 3 h after administration of the first maintenance doses (Fig. 5a); additionally, body temperature drop was less observed after administration of the rest maintenance doses (data not shown). Moreover, the mice that displayed severe temperature drop (≥ 1 °C) were also lethargic and were at obviously increased risk of treatment-related-death (Fig. 5b). Mouse death mostly occurred involving the administration of BC31M5 or Hu5F9 in doses higher than 10 mg/kg (Fig. 5b).

When mice were directly treated with a high dose (20 mg/kg) of antibody *i.p.* without priming, both BC31M5 and Hu5F9 caused severe body temperature drop in more than half of the mice, and this was accompanied with very high treatment-related-death rates (≥ 50%); however, similar side effects occurred in only 6% (1/16) of the BC31M4-treated mice. After adopting the prime-plus-maintenance treatment strategy comprising a priming dose of 1 mg/kg and maintenances doses of 10 mg/kg, no side effects were observed in the BC31M4 treated mice. In contrast, the proportions of mice displaying severe body temperature drop and/or treatment-related-death rates were still very high upon either BC31M5 (38%) or Hu5F9 (29%) treatment under the maintenance dose of 10 mg/kg. When the maintenance dose was further decreased to 1 mg/kg, no treatment-related-death occurred for Hu5F9-treated mice, but 1 out of 10 animals displayed a severe temperature drop. Moreover, when antibodies were converted into the mIgG1 isotype (weak Fc effector functions), neither BC31M4-mIgG1 nor Hu5F9-mIgG1 caused any side effects in mice treated with maintenance doses of 10 mg/kg (Fig. 5a, b). These results suggest that the tolerability of anti-CD47 antibody is Fc-effector function dependent.

As a consequence of the relatively high expression of CD47 on RBCs and platelets, hematotoxicity—which includes anemia, thrombocytopenia, hemagglutination, and neutropenia—has been a major concern among the various side effects observed in clinical studies of anti-CD47 therapies [16–18]. We next sought to determine the basis of the severe side effects observed in mice during antibody treatment and further evaluate the safety of BC31M4. We first performed an in vitro hemagglutination assay using RBCs from the C57-hCD47/hSIRPα mice. The RBC aggregation was further assessed by flow cytometry. Compared to Hu5F9, BC31M4 caused obviously less RBC aggregation, represented by the lower percentage of cell populations with high FSC-A and SSC-A values (Fig. 5c).

We next evaluated the in vivo hematotoxicity of BC31M4. Given that both BC31M5 and Hu5F9 caused severe side effects (to a similar extent) (Fig. 5a, b), we selected Hu5F9 for comparison in the following assay. Healthy mice were treated with a single dose of 20 mg/kg antibodies (mIgG2a isotype) or PBS *i.p*., and hematologic parameters were assessed at 3 h after treatment. Compared to the PBS control, we detected significant decreases in RBC count, hemoglobin (Hgb), and hematocrit (HCT) in mice treated with Hu5F9 (Fig. 5d and Additional file 1: Fig. S4a, b), features indicating acute anemia; and the RBC distribution width (RDW) was significantly increased (Fig. 5e), indicating increased variation in RBC volume that could result from RBC aggregation. This was consistent with the above finding that Hu5F9 caused more severe RBC aggregation than BC31M4 in vitro. In contrast, mice treated with BC31M4 did not exhibit any alteration in these indices. Besides, the significant increase of the spleen weight of mice treated with Hu5F9 (Fig. 5f) is consistent with the commonly observed splenomegaly in hemolytic anemia due to accumulation of macrophages and CD47^−/−^ RBCs in mice [1].

Significant drops in platelet counts were observed in mice treated with BC31M4 and Hu5F9 (Fig. 5g). However, a significant increase in mean platelet volume (MPV, indicating platelet aggregation) was observed for Hu5F9-treated but not for BC31M4-treated mice (Fig. 5h). Moreover, compared to the proportion of individual leukocytes in PBS controls, BC31M4 treatment did not change the proportion of individual leukocytes assessed; whereas Hu5F9 treatment caused a significant decrease in neutrophils (~ 22%) that was accompanied by relative increases in lymphocytes and basophils, which indicated neutropenia of Hu5F9-treated mice (Fig. 5i). Collectively, these results showed that BC31M4 causes minimal hematotoxicity during treatment.

### BC31M4 efficiently promotes adaptive immune responses against tumors in syngeneic mouse models

We next evaluated the therapeutic efficacy of antibodies in syngeneic mouse models. The anti-CD47 antibodies (mIgG2a isotype) alone did not exert any antitumor effect in the syngeneic mouse models (Additional file 1: Fig. S5a–e). Previous studies showed that the activation of the adaptive immune system was required for the antitumor effect of anti-CD47 therapy in immunocompetent syngeneic mouse models [21, 22]. Thus, we explored whether BC31M4 promotes antitumor responses against syngeneic tumors in mice with a stimulated adaptive immune system (combination therapy). The E.G7 cell line is derived from EL4 lymphoma cells and expresses ovalbumin (OVA); the OT-I T cells are OVA-specific CD8^+^ T cells and can be isolated from OT-I transgenic mice [41]. To determine whether Fc effector function is required in this combination therapy, BC31M4 or Hu5F9 was also converted into mIgG1 isotype and included for evaluation in these experiments.

**Fig. 6:**
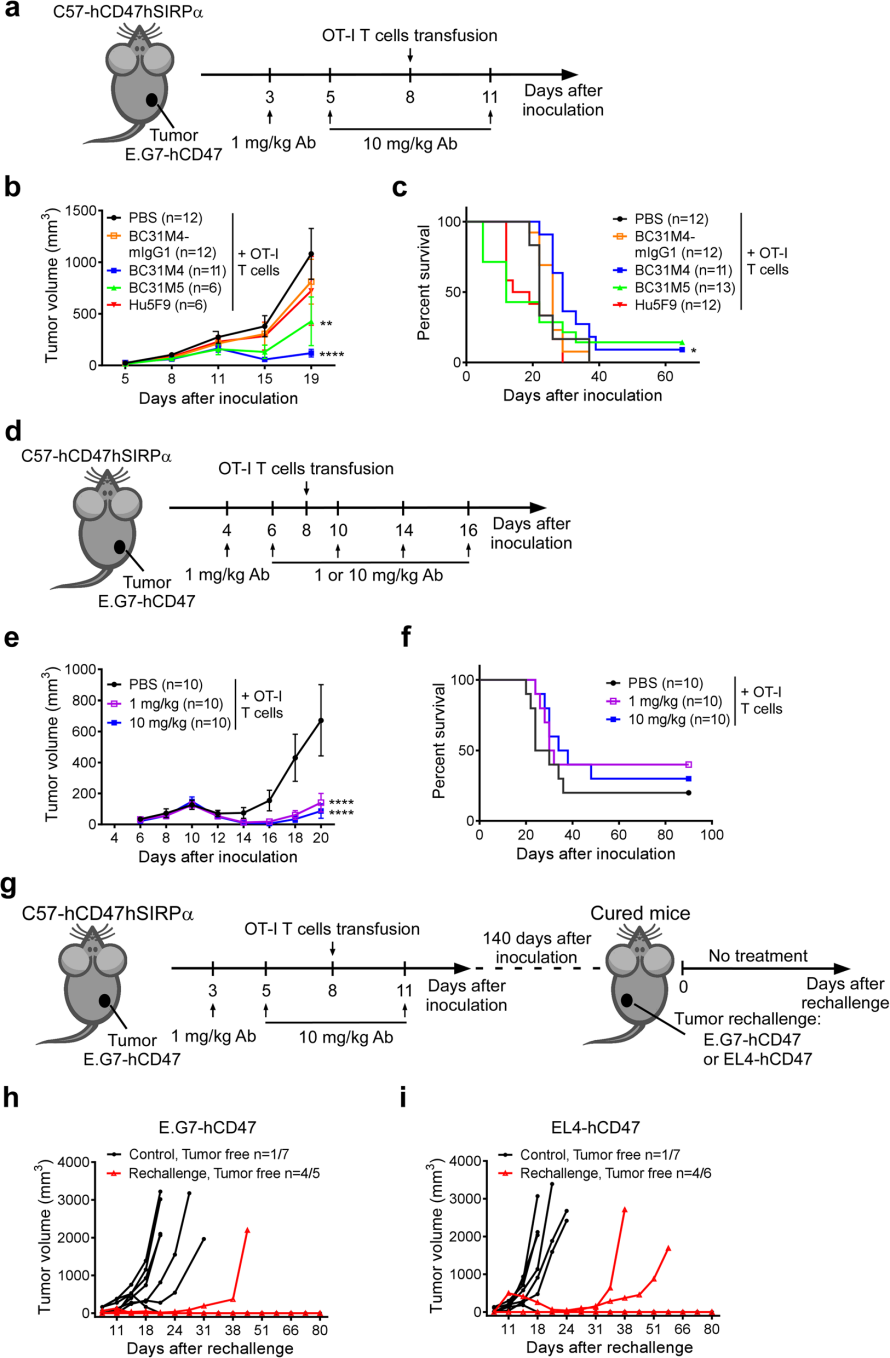
BC31M4 efficiently promotes the adaptive immune responses against tumors in syngeneic mouse models and promotes the development of immune memory. **a** Schematic diagram of tumor inoculation and treatment. C57-hCD47/hSIRPα mice were *s.c.* inoculated with E.G7-hCD47 cells on the right lower flank. Antibodies or PBS were *i.p.* injected, OT-I T cells were *i.v.* transfused. **b** The tumor growth of mice treated as in (**a**); Treatment-related-death mice were excluded from summary statistics. **c** Survival of mice in (**b**). **d** Schematic diagram of tumor inoculation and treatment. **e** The tumor growth of mice treated as in (**d**). **f** Survival of mice in (**e**). **g** Schematic diagram of tumor rechallenge. C57-hCD47/hSIRPα mice were initially inoculated and treated as in (**a**), cured mice were rechallenged with E.G7-hCD47 or EL4-hCD47 cells about 140 days after the initial tumor inoculation. The rechallenged tumor cells were *s.c.* inoculated on the left lower flank. **h**, **i** Tumor growth in rechallenged mice is shown individually. Age-matched, naïve, C57-hCD47/hSIRPα mice were included as controls. Proportions of tumor free mice are indicated. n, number of mice. Antibodies used in (**a–g**) are mIgG2a isotype unless otherwise noted

We subsequently established the E.G7-hCD47 syngeneic models as described above, and treated these mice *i.p.* with a priming 1 mg/kg antibody dose (or PBS) on day 3, followed by two maintenance doses of 10 mg/kg on day 5 and 11; OT-I T cells were intravenously (*i.v.*) transfused into all mice on day 8 (Fig. 6a). Compared to the OT-I T cell monotherapy (i.e., PBS priming and maintenance), the BC31M4 and OT-I T cell combination therapy significantly delayed tumor growth and significantly prolonged the survival of mice. Although BC31M5 also significantly inhibited tumor growth compared to the OT-I T cell monotherapy, it is not as potent as BC31M4, which reflects its decreased tumor selectivity and faster PK than BC31M4 in vivo. Furthermore, combination therapies using the Hu5F9 or BC31M4-mIgG1 did not affect tumor growth and survival (Fig. 6b, c). We also observed in a similar combination therapy experiment that Hu5F9-mIgG1 did not confer any antitumor effects (Additional file 1: Fig. S6). These results suggest that BC31M4 is more potent than the other antibodies in promoting adaptive antitumor immune responses in syngeneic mouse models, and the antitumor activity of BC31M4 depends on the Fc effector function.

Recalling that BC31M4 exhibited intratumoral accumulation after the 1 mg/kg priming dose (Additional file 1: Fig. S3d), it is possible that, deployed as combination therapy, low dosages of BC31M4 may confer similarly potent antitumor activity as high dosages. To test this proposition, the E.G7-hCD47 lymphoma model was treated with a combination of BC31M4 and OT-I T cells in a similar manner as described above. Mice were treated *i.p*. with a priming 1 mg/kg BC31M4 (or PBS) on day 4, followed by 4 maintenance doses of 1 or 10 mg/kg on days 6, 10, 14, and 18. OT-I T cells were transfused into all mice on day 8 (Fig. 6d). Strikingly, compared to the OT-I T cell monotherapy, the antitumor efficacy under the maintenance doses of 1 mg/kg was as potent as the maintenance doses of 10 mg/kg, both of which significantly inhibited tumor growth (Fig. 6e, f). These results suggest that the tumor selectivity of BC31M4 confers it with high efficiency in promoting the antitumor immune responses in syngeneic mouse models.

### BC31M4 promotes the activation of native T cells and the development of immune memory

We next sought to determine whether the surviving mice after the combination therapy developed immune memory. Mice that survived a similar experiment—in which E.G7-hCD47 lymphoma model animals were treated with the combination of anti-CD47 antibody and OT-I T cells—were rechallenged with the E.G7-hCD47 cells or the parental EL4-hCD47 cells that did not express OVA, about 4 months after the initial tumor challenge (Fig. 6g). Previously untreated age-matched C57-hCD47/hSIRPα mice were challenged with the same tumor cells as controls.

All survived mice were resistant to the either E.G7-hCD47 rechallenge or EL4-hCD47 rechallenge. Although tumor growth was observed in one (1/5) mouse rechallenged with E.G7-hCD47 and two (2/6) mice rechallenged with EL4-hCD47 cells, the growth was much slower than in control mice, and most of the survived mice completely rejected the rechallenge cells. In contrast, in the control mice, rapid tumor growth was observed in six (6/7) mice challenged with E.G7-hCD47 and six (6/7) mice challenged with EL4-hCD47, (Fig. 6h, i). These results suggest that most of the survived mice have immune memory. Moreover, these results indicate that most of the memory cells were specific to the antigen(s) common in E.G7-hCD47 and EL4-hCD47 cells, rather than the dominant antigen OVA in E.G7-hCD47 cells, a finding implicating the activation of native T cells in the observed immune responses. Collectively, these results suggest that BC31M4 promotes the activation of native T cells against tumors and the development of immune memory in syngeneic mouse models.

## Discussion

The overexpression of antiphagocytic molecule CD47 on various tumor cells has made it a promising therapeutic target. However, the ubiquitous expression of CD47 on healthy cells poses a substantial hurdle for the development of safe and effective anti-CD47 therapies. In the present study, we aimed to overcome this dilemma by improving the tumor selectivity of anti-CD47 antibodies, and our approach was to exploit the known acidic microenvironment of solid tumors. We developed a pH-dependent anti-CD47 antibody that selectively binds to cells in solid tumors but sparing cells in normal tissues in immunocompetent syngeneic mouse models, which exhibits a favorable safety profile. When combined with adoptive T cell transfer, BC31M4 efficiently promotes adaptive antitumor immune responses as well as the development of immune memory.

Anti-CD47 antibodies have exhibited potent antitumor efficacy in many human tumor xenograft models, specifically by promoting the tumoricidal activity of macrophages [3], and similar results were observed in our study. However, several limitations of using xenograft models to study anti-CD47 therapy have been highlighted in previous studies [21, 22]. The mice used in these xenograft models are immunocompromised, lacking adaptive immune function but retaining functional macrophages that are responsible for the antitumor effects under CD47 blockade. It is therefore highly notable that the antibodies examined in these studies only target tumor cells expressing human CD47 in these models. Moreover, almost all antibodies used in these models are known to mediate strong Fc effector function that potentiates the antitumor activity. As a consequence, the antitumor efficacy of these antibodies may have been overestimated; and the treatment-related side effects caused by binding with CD47 on healthy cells has almost certainly been overlooked and/or underestimated. Consistent with this notion, we found that BC31M4, BC31M5 and Hu5F9 examined in this study confer potent antitumor efficacy in xenograft models without any side effects. However, these antibodies did not exhibit antitumor effect in syngeneic mouse models.

Hematotoxicity is a major concern with anti-CD47 therapies. We found that several of the side effects reported from the phase I study of Hu5F9, were also observed in C57-hCD47/hSIRPα mice, such as anemia, thrombocytopenia, hemagglutination, neutropenia, and chills (severe body temperature drop) [16, 17]. Several studies have reported the development of anti-CD47 antibodies (or SIRPα-Fc fusion protein) that bind minimally to CD47 on the surface of RBCs or some other normal cells, which have exhibited good safety profiles when tested in preclinical and clinical studies [42–44], However, it is noteworthy that CD47 is also ubiquitously expressed on normal tissues, so the impact(s) of anti-CD47 agents on other healthy cells should be thoroughly investigated. In this study, we showed that BC31M4 exhibits favorable safety profile owing to its selective binding to cells in solid tumors in immunocompetent syngeneic mouse models.

It has been demonstrated that blockade of the CD47/SIRPα signaling alone is insufficient to inhibit tumor growth in the absence of additional pro-phagocytic signals (*e.g.*, Fc-FcγR-mediated effector function). Similarly, our in vitro phagocytosis analysis results demonstrated that the Fc-mediated effector functions of BC31M4 is required to promote macrophages phagocytosis of tumor cells. Accordingly, most of the antibodies examined in the present study were in hIgG1 or mIgG2a isotypes that mediate strong Fc effector function. We did observe in vivo that BC31M4 in mIgG2a isotype efficiently promoted antitumor immunity when combined with adoptive T cell transfer; however, conversion of BC31M4 into the mIgG1 isotype (thereby reducing the strength of its Fc effector function) abrogated its antitumor effects. Our results suggest that strong Fc effector function is required to maximize the antitumor efficacy of anti-CD47 therapy in immunocompetent hosts. However, paradoxically, strong Fc effector function could lead to severe side effects. In our study, the side effects of Hu5F9 were more severe in mice than those observed in patients, which may be a result of the different isotypes used. Specifically, the Hu5F9 used in patients is the hIgG4 isotype, which mediates weak Fc effector function [37], while the Hu5F9 used in mice herein was the mIgG2a isotype. We observed no side effects when Hu5F9 was converted into mIgG1. This may explain reports from clinical trials indicating that most of the anti-CD47 agents with weak Fc effector function can be tolerated in patients [16, 46, 47].

Although a few studies have shown that an anti-CD47 antibody monotherapy was able to inhibit tumor growth in some syngeneic mouse models [21], most studies in syngeneic mouse models and in clinical trials reported that anti-CD47 antibody alone did not exert significant antitumor effects [16, 22, 48, 49]. Consistently, our data also showed that anti-CD47 antibodies (including BC31M4, BC31M5 and Hu5F9) monotherapy did not confer antitumor activity in the syngeneic mouse tumor models although they conferred potent antitumor activity in xenograft models. Moreover, most of the recent clinical trials involving anti-CD47 agents are examining combination modalities that include other antitumor agents. Several studies in syngeneic mouse models have reported that stimulation of adaptive immune responses is required to obtain an antitumor benefit from certain anti-CD47 therapies [21, 22]. Consistently, we found that BC31M4 efficiently promoted antitumor immunity of both transferred T cells and native T cells when combined with adoptive T cell transfer. We speculate that BC31M4 promoted the phagocytosis of tumor cells through blocking the CD47-SIRPα interaction and engaging the activating FcγRs on phagocytes, after which such phagocytes may be activated to prime T cell immunity and to induce immune memory. However, BC31M4 monotherapy did not exhibit antitumor effects, perhaps owing to the inhibited and/or exhausted phenotypes of T cells in the tumor microenvironment [50]. Accordingly, combining BC31M4 with additional immune-modulating agents to activate antitumor T cells in tumors may be an attractive approach for future development.

Comparing BC31M4 (high pH-dependence), BC31M5 (weak pH-dependence) and Hu5F9 (no pH-dependence), the absolute accumulation of BC31M4 in tumors is much higher than BC31M5 and Hu5F9, which might have contributed to the better antitumor effect of BC31M4 when combined with adoptive T cells transfer in syngeneic mouse models. Temporarily disregarding BC31M5’s severe side effects, it is noteworthy that although BC31M5 and Hu5F9 have similarly poor PK properties, BC31M5 exhibited higher antitumor efficacy than Hu5F9 when combined with adoptive T cell transfer. Given the weak pH-dependence of BC31M5, BC31M5 still exhibits higher relative intratumoral accumulation than Hu5F9, highlighting the apparently advantage of tumor selectivity for anti-CD47 therapy. Several studies have attempted to improve the tumor selectivity of anti-CD47 agents by generating bispecific antibodies (i.e., an antibody which recognizes two different antigens on tumor cells simultaneously), which have been demonstrated to minimize side effects [34, 51]. Moreover, a recent study sought to improve therapeutic efficacy safety for ovarian cancer by engineered an oncolytic herpesvirus to express anti-CD47 antibodies in tumors [52]. However, the selectivity of these antibodies is restricted to tumors that expressed two specific targets. As to BC31M4 we developed, its pH-dependent binding relies on the existence of an acidic condition in tumors. Although solid tumors often have a pH in the range of 6.4–6.8, pH can vary from 5.8–7.6 depending on tumor type, size, location, and metabolic state [28, 53, 54]. Thus, the clinical application of BC31M4 would be for solid tumors with an acidic microenvironment.

Several studies have reported the generation of “reversed” pH-dependent antibodies, which bind to antigen with high affinity at physiological-pH but with low affinity at acidic-pH; these antibodies are generated by introducing histidines into the variable regions of the corresponding parental antibodies [55–58]. A recent study reported the generation of a pH-dependent anti-human Her2 antibody that has selective binding in acidic conditions (again based on introducing histidines into the parental antibody), and demonstrated tumor inhibition ability only in vitro [59]. In our study, we generated BC31M4 with high pH-dependence by employing antibody phage display technology and a pH-dependent selection strategy. By solving the co-crystal structure of CD47 and its close variant antibody BC31M5, and site saturation mutagenesis of histidines in the CDRs of BC31M4, we determined the structural basis of the pH-dependent binding property of BC31M4. Histidines H38 and H107 in CDRs of the light chain contribute to the pH-dependent binding of BC31M4, which rely largely on their protonation state switch that occurs around pH 6.8. BC31M4 and BC31M5 have only one amino acid (A108 in BC31M4, and T108 in BC31M5) difference, but the pH-dependence of BC31M4 is higher (about 22-fold as examined by SPR) than BC31M5; the weak pH-dependence of BC31M5 is likely due to the strong polar contacts formed by T108, which can apparently compensate for the loss of electrostatic contacts formed by H38 and H107 at physiological-pH, as evidenced by the higher affinity of BC31M5 at pH 7.4.

Many anti-CD47 antibodies are currently under preclinical investigations and clinical trials. The major challenges for these antibodies are the side effects associated with CD47 blockade and the weak therapeutic efficacy in solid tumors. There are other challenges, including tumor heterogeneity and an immunosuppressive tumor microenvironment. Our results suggest that, compared to an anti-CD47 monotherapy, combination therapies designed both to selectively target tumor cell killing and to promote adaptive immune responses should be more efficacious for treating solid tumors in patients. Our development of a tumor-selective, pH-dependent anti-CD47 antibody confirms that the acidic tumor microenvironment is an exploitable characteristic for effective deployment of antibodies to treat solid tumors. More generally, our study illustrates a strategy for generating antibodies against solid tumor antigens that are also expressed by healthy tissues.

## Conclusion

Our study successfully developed a tumor-selective, pH-dependent anti-CD47 antibody (BC31M4) that safely confers strong therapeutic effects against solid tumors. Additionally, our results demonstrated that BC31M4’s Fc effector function is required for the antitumor activity. These results illustrate how improving the tumor selectivity of a therapeutic anti-CD47 antibody while also promoting Fc-FcγR-mediated effector function can safely enhance antitumor efficacy in immunocompetent hosts. Our work thus provides a promising therapeutic strategy to overcome the challenges regarding the therapeutic efficacy and safety of anti-CD47 therapies.

## Supplementary Information

**Additional file 1:** Supplementary table (Table S1) and figures (Figs. S1–S6).

## Data Availability

All relevant data are included in this manuscript and its supplementary information files. Structure information will be released from the Protein Data Bank (PDB ID 7WN8) upon publication of this manuscript.

## Abbreviations

SIRPα: Signal regulatory protein alpha
RBC: Red blood cell
Fc: Fragment crystallizable
FcγR: Fc gamma receptor
PK: Pharmacokinetic
OVA: Ovalbumin
ECD: Extracellular domain
mIgG2a: Mouse IgG2a
hIgG1: Human IgG1
mIgG1: Mouse IgG1
scFv: Single-chain variable fragment
CDR: Complementarity-determining region
SPR: Surface plasmon resonance
K_D_: Equilibrium dissociation constant
ELISA: Enzyme-linked immunosorbent assay
EC_50_: Half maximal effective concentration
IC_50_: Half maximal inhibitory concentration
ADCP: Antibody-dependent cellular phagocytosis
BMDM: Bone marrow-derived macrophage
