# Supplementary material for: A pH-dependent anti-CD47 antibody that selectively targets solid tumors and improves therapeutic efficacy and safety
Source: J Hematol Oncol. 2023 Jan 17;16:2. doi: 10.1186/s13045-023-01399-4 (PMC9844003; doi:10.1186/s13045-023-01399-4)
Supplement: Supplementary file 1 — Additional file 1: Supplementary table (Table S1) and figures (Figs. S1–S6). [file 13045_2023_1399_MOESM1_ESM.docx]

| **Complex** | **BC31M5-CD47** |
| --- | --- |
| **Data collection** |  |
| Wavelength (Å) | 0.9793 |
| Resolution range (Å) | 49.59 - 2.80 (2.901 - 2.801) |
| Space group | P2_1_ 2_1_ 2_1_ |
| Unit cell dimensions |  |
| a, b, c (Å) | 91.402, 94.281, 174.929 |
| α, β, γ (°) | 90, 90, 90 |
| Multiplicity | 13.2 (11.8) |
| No. of unique reflections measured | 37875 (3664) |
| Completeness (%) | 99.76 (98.28) |
| CC_1/2_ (%) | 0.997 (0.848) |
| R_merge_ (I) | 0.1613 (0.94) |
| I/σ(I) | 16.96 (2.82) |
|  |  |
| **Refinement** |  |
| Resolution (Å) | 49.59 - 2.80 (2.87 - 2.80) |
| Reflections used in refinement | 37870 (3664) |
| Reflections used for R-free | 1998 (187) |
| R-work | 0.2434 (0.3780) |
| R-free | 0.2759 (0.4059) |
| No. non-hydrogen atoms | 8402 |
| RMS(bonds) (Å) | 0.003 |
| RMS(angles) (°) | 0.66 |
| Mean B value (Å^2^) | 44.6751 |
| Ramachandran favored (%) | 99.72 |
| Ramachandran allowed (%) | 2.09 |
| Ramachandran outliers (%) | 0.19 |

**Additional file 1—Supplementary Table and Figures**

This file includes 1 supplementary table and 6 supplementary figures.

**Table S1** **Data collection and refinement statistics**

Values in parentheses are for highest-resolution shell.

**
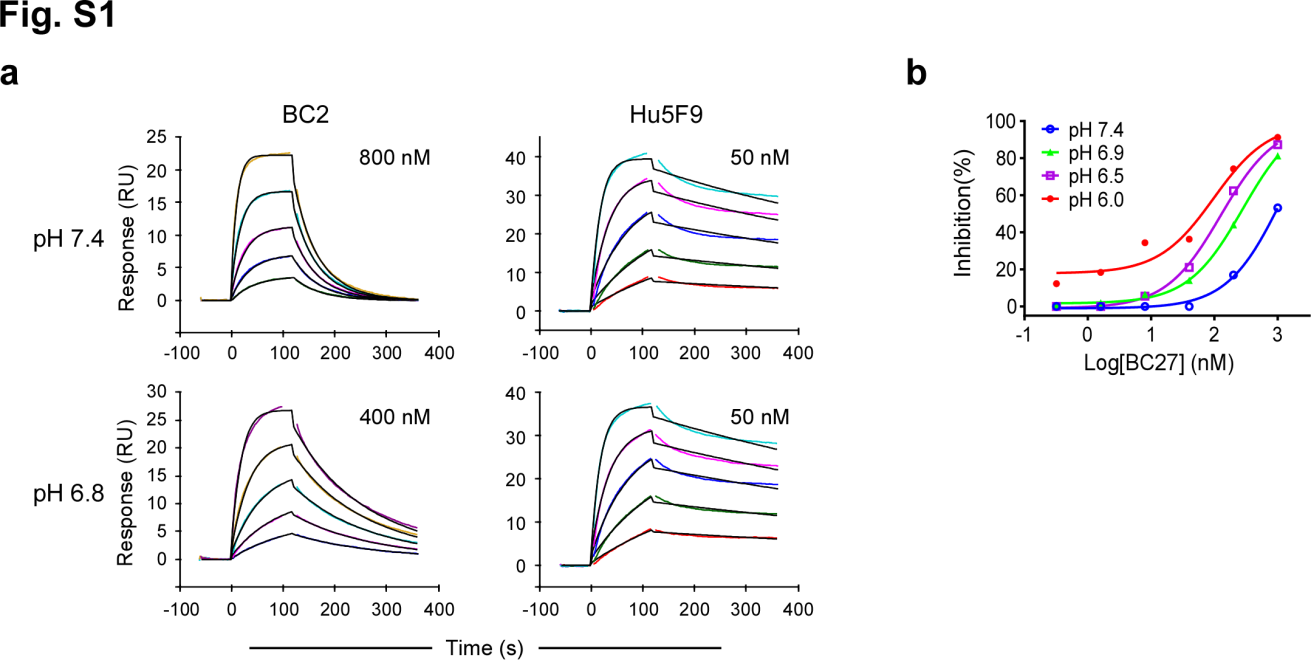
**

**Fig. S1** Characterization of pH-dependent anti-CD47 antibodies. **a** Representative SPR sensograms of BC2 and Hu5F9. **b** BC27 blocks SIRPα binding to the cell-surface CD47. Bio-SIRPα-Fc binding to CHO-hCD47 in the presence of serial dilutions of BC27, measured by flow cytometry. The blocking activity of antibodies is shown as the percentage of inhibition by normalizing the value of ‘bio-SIRPα-Fc only’ as 0% inhibition. This assay was performed at pH 7.4, pH 6.9, pH 6.5, and pH 6.0.


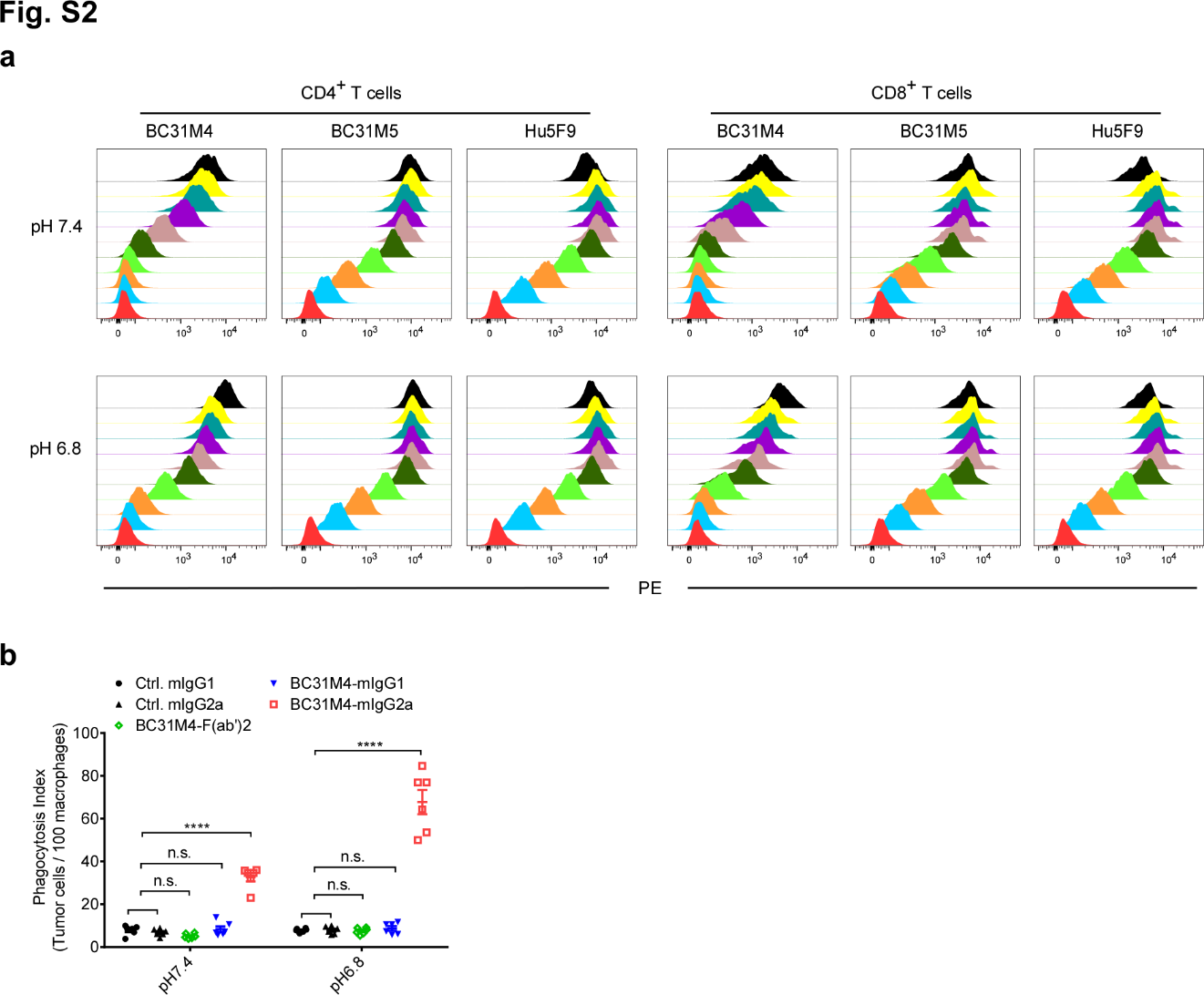


**Fig. S2** BC31M4 binds to cell-surface CD47 and promotes macrophage phagocytosis of tumor cells. **a** Anti-CD47 antibodies binding to normal T cells. Serially diluted antibodies (in hIgG1 form) binding to human CD4^+^ and CD8^+^ T cells in PBMCs at pH 6.8 and 7.4, analyzed by flow cytometry. Antibodies are 4-fold serially diluted from 500 nM. **b** BC31M4 promotes macrophages phagocytosis of tumor cells. Macrophages phagocytosis of E.G7-hCD47 cells under different antibody treatments at pH 7.4 and pH 6.8. Antibody concentration is 10 μg/ml. Phagocytosis index is determined as the number of phagocytosed E.G7-hCD47 cells per 100 macrophages.

.


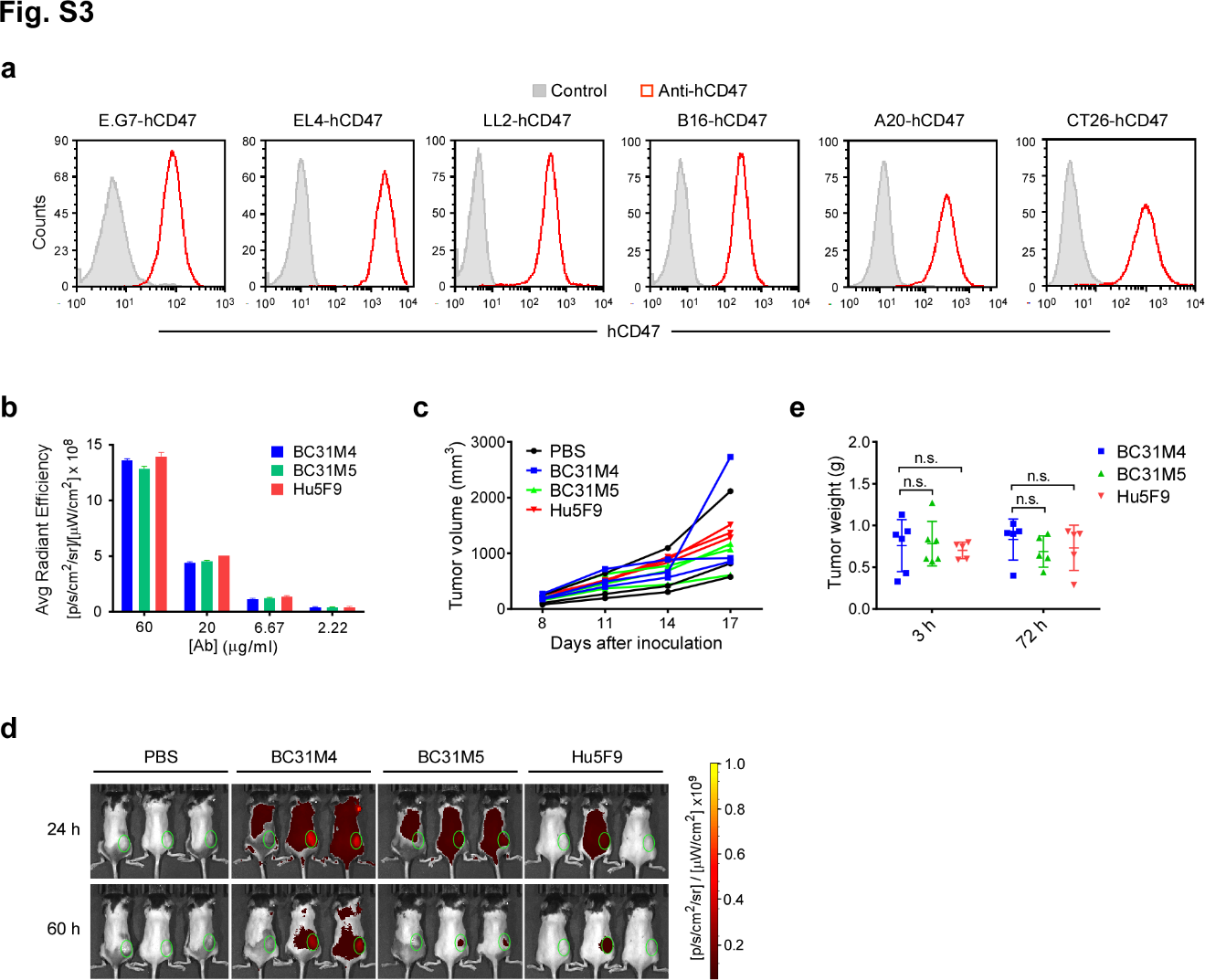


**Fig. S3** Evaluation of antibody distribution in syngeneic mouse tumor models. **a** Expression of hCD47 on the surface of humanized mouse tumor cell lines. The indicated tumor cell lines were stably transfected with full-length hCD47. Cells were incubated with hCD47-specific antibodies, followed by detection using a goat anti-human IgG-FITC antibody, analyzed by ﬂow cytometry. **b** Evaluation of the average radiant efficiency of the labeled antibodies. Antibodies were serially diluted in a 96-well white plate for fluorescence imaging using the IVIS Lumina III Imaging System with excitation at 745 nm and emission measured at 800 nm, and the average radiant efficiency was quantified using the Living Image Analysis Software. Data are shown as the mean ± SEM. **c** Tumor growth in mice of Fig. 4b is shown individually. **d** Fluorescence imaging of mice in Fig. 4b at 24 h and 60 h after the priming dose of 1 mg/kg. Green circles indicate the location of tumors. **e** Weight of tumors in Fig. 4c–e.


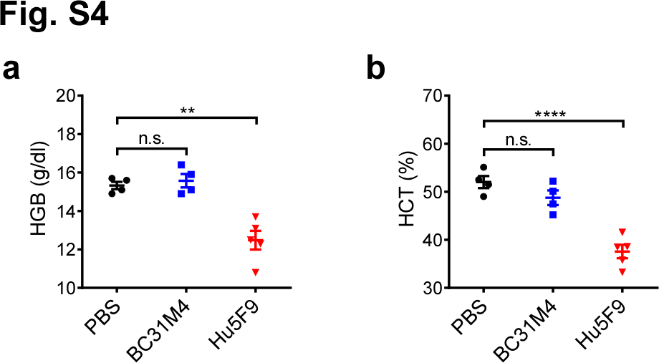


**Fig. S4** BC31M4 causes minimal hematotoxicity in C57-hCD47/hSIRPα mice. **a**, **b** Part of the complete blood count test results of antibody treated mice in Fig. 5d–i. The parameters of hemoglobin (**a**) and hematocrit (**b**) are shown. Antibodies used are mIgG2a isotype.


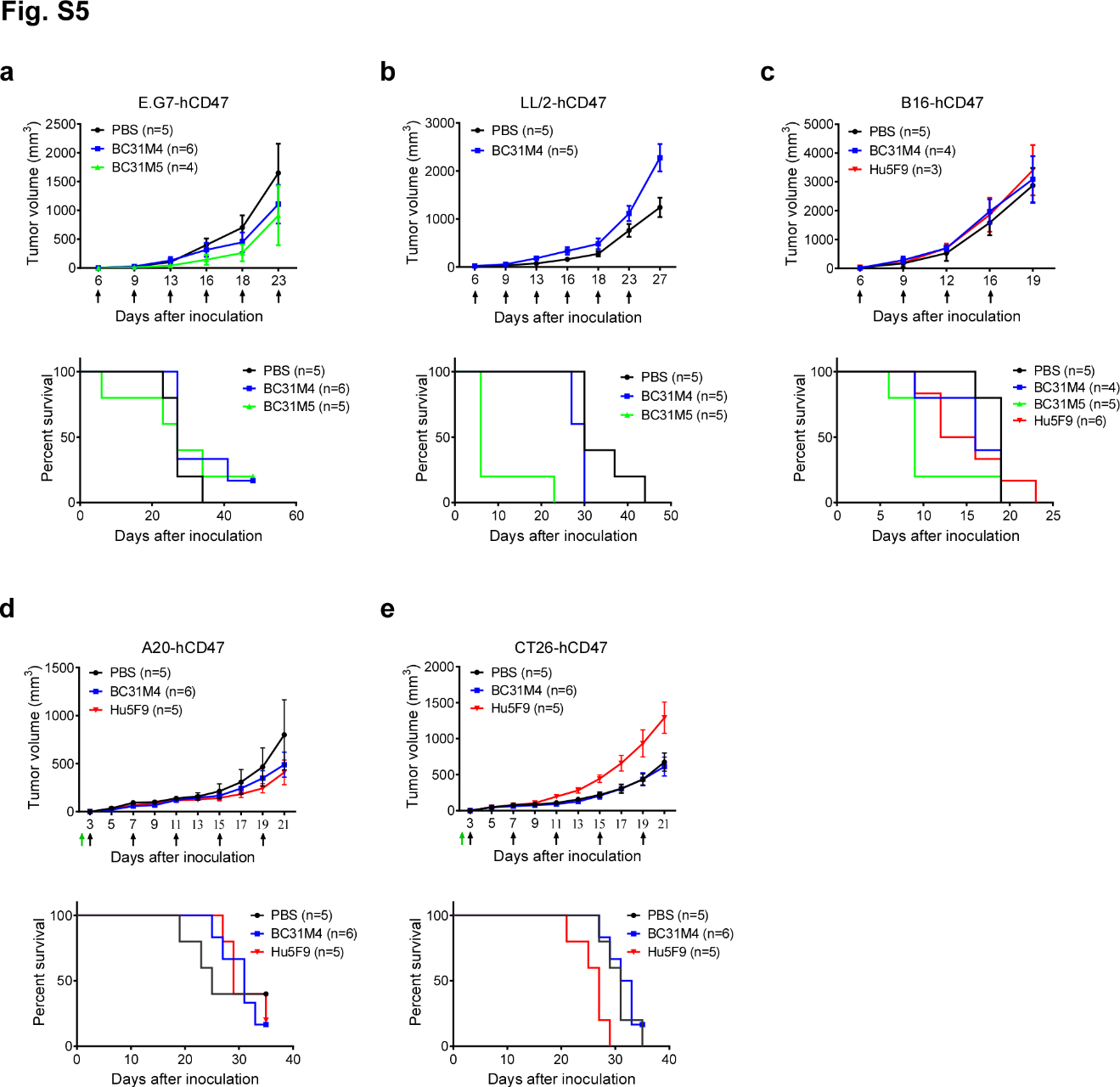


**Fig. S5** Anti-CD47 antibody monotherapy does not confer an antitumor effect in syngeneic mouse models. **a**–**c** Antitumor activity of antibodies in C57-hCD47/hSIRPα based syngeneic mouse models. C57-hCD47/hSIRPα mice were *s.c.* inoculated with E.G7-hCD47 (**a**), LL/2-hCD47 (**b**), or B16-hCD47 (**c**) tumor cells, and *i.p.* treated with 20 mg/kg antibodies or PBS. Black arrows indicate antibody treatment. Tumor growth (top) and survival (bottom) of mice are shown. Mice that died of treatment-related side effects were excluded from the tumor growth summary statistics. Tumor growth of the BC31M5-treated groups in (**b, c**) is not shown, owing to the high rate of treatment-related deaths. **d**, **e** Antitumor activity of antibodies in BALB/c-hCD47/hSIRPα based syngeneic mouse models. BALB/c-hCD47/hSIRPα mice were *s.c.* inoculated with A20-hCD47 (**d**) or CT26-hCD47 (**e**) tumor cells. Mice were *i.p.* injected with a priming dose (green arrows) of antibodies (1 mg/kg) or PBS as control, followed by maintenance doses (10 mg/kg, black arrows). Tumor growth (*top*) and survival (*bottom*) of mice are shown. n, number of mice. Antibodies used in (**a–e**) are mIgG2a isotype.


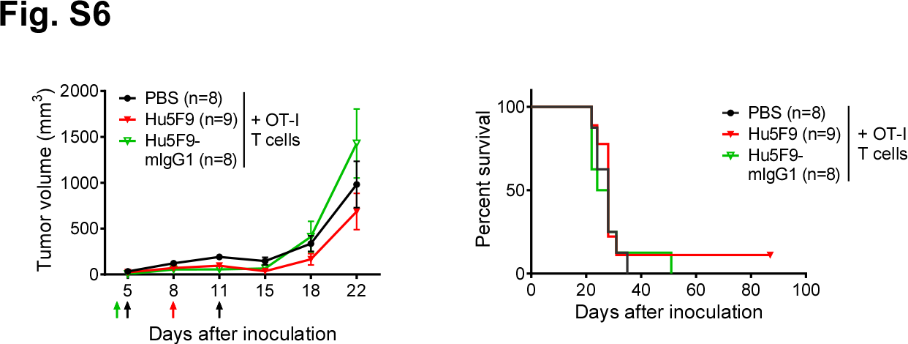


**Fig. S6** Antitumor activity of anti-CD47 antibody in combination with adoptive T cell transfer in syngeneic mouse models. C57-hCD47/hSIRPα mice were *s.c.* inoculated with E.G7-hCD47 cells. Mice were *i.p.* injected with a priming dose of antibodies (1 mg/kg, green arrows) or PBS, followed by maintenance doses (10 mg/kg, black arrows). OT-I T cells were *i.v.* transfused (red arrow). Tumor growth (*left*) and survival (*right*) are shown. n, number of mice. Antibodies used are mIgG2a isotype unless otherwise noted.
